# Parkinson’s Disease in Romania: A Scoping Review

**DOI:** 10.3390/brainsci11060709

**Published:** 2021-05-27

**Authors:** Elena Cecilia Rosca, Raluca Tudor, Amalia Cornea, Mihaela Simu

**Affiliations:** 1 Department of Neurology, Victor Babes University of Medicine and Pharmacy of Timisoara, Eftimie Murgu Sq. No. 2, 300041 Timișoara, Romania; tudor.raluca@yahoo.com (R.T.); amalia.cornea@yahoo.com (A.C.); mihaelasimu6713@gmail.com (M.S.); 2 Department of Neurology, Clinical Emergency County Hospital Timisoara, Bd. Iosif Bulbuca No. 10, 300736 Timisoara, Romania; 3 Neuroscience Research Center Timisoara, Clinical Emergency County Hospital Timisoara, Bd. Iosif Bulbuca No. 10, 300736 Timisoara, Romania

**Keywords:** Parkinson’s disease, Romania, scoping review

## Abstract

Parkinson’s disease (PD) is a significant cause of disability, with a fast-growing prevalence. This review summarizes the epidemiological and clinical data, research on the diagnostic approaches and the interventions available in the Eastern European country of Romania. This scoping review follows the recommendations on the scoping review methodology by Joanna Briggs Institute. We searched four databases (up to 27 January 2021). The data of eligible studies were extracted in standardized forms. We identified 149 unique studies from 1133 records, with 11 epidemiological studies, 52 studies investigating clinical aspects of PD, 35 studies on diagnostic tools, and 51 intervention studies. A narrative synthesis is provided and placed in a historical context. Our review revealed a considerable increase in the Romanian research on PD in the latest 15 years, which largely follows international trends. However, we also identified several research gaps that provide useful information for policymakers, public health specialists, and clinicians.

## 1. Introduction

Parkinson’s disease (PD) is a neurodegenerative disorder reported to be a leading global cause of disability. Among the neurological disorders, it was found to present the fastest growth in prevalence, disability, and deaths [1]. Namely, in 2016, the overall worldwide number of individuals with PD was 2.4 times higher than in 1990. A systematic analysis of epidemiological studies estimated that 6.1 million people worldwide had PD; the disease caused 211,296 deaths and 3.2 million disability-adjusted life-years (DALYs). Furthermore, it is estimated that the number of PD cases will double in the coming generation due to increasing life expectancy.

To understand the current situation of PD in Romania, it is essential to identify all the available data and map the severity of the problem accurately. A clear presentation of the research results and trends provides useful insight into the country’s context, assisting researchers, healthcare professionals, and policymakers in decision-making. In addition, it will guide the development of future research strategies, and it will help the design and implementation of programs to reduce the burden of PD in Romania [2].

Consequently, to address this burden, there is a need for effective treatment and care strategies. In addition, data on the incidence and prevalence of the disease, especially in regions in which little data are available, are essential [1].

Romania is a middle-income, formerly socialist Eastern European country, with approximately 19 million people, 17.8% over 65 years old. The patient’s association, a non-governmental organization, estimates there are over 72,000 patients with PD in Romania [3]. This estimation is based on the number of PD medication prescriptions reported by the National Health Insurance House. Nonetheless, some patients with Parkinsonian syndromes may also respond to the PD medication; therefore, this report probably overestimates the number of PD patients in Romania. Furthermore, a systematic review estimating global, regional, and country-specific data on PD reports that in Romania, in 2016, there were 40,517 (95% uncertainty interval UI 31,427 to 50,995) patients with PD, with 23,144 (95% UI 17,467 to 30,057) disability-adjusted life-years (DALY’s) [1].

A study estimating the cost of disorders of the brain in Europe [4] reported that in Romania, in 2010, there were 43,841 individuals with PD; the costs per person for these patients was 3933 Euros purchasing power parities (PPPs). The highest costs in Europe were reported in Luxembourg and United Kingdom, with 21,475 Euros PPP, respectively 21,500 Euros PPP per PD patient. The lowest costs were reported in Bulgaria, with 3143 Euros PPP per PD patient, followed by Romania [4]. These total costs include direct costs (healthcare costs and non-medical costs) and indirect costs associated with patients’ production losses. All the costs are expressed in PPPs adjusted real EUR. The estimates presented in the review were converted to real EUR, using nominal exchange rates from the European central bank (ECB), adjusted for comparative price levels (CPL) for 2009 from Eurostat. The CPL was defined as the ratio of purchasing power parities to exchange rates. The selected CPLs were based on the total consumption in each country (GDP). For example, they were not limited to price differences in healthcare goods [4].

The data from Eurostat show that in 2018, Romania had a physician/inhabitant ratio of 254.99/100,000 and a hospital bed/inhabitant ratio of 669.83/100,000. The Romanian Society of Neurology estimates that currently, there are approximately 750 practicing neurologists.

The present scoping review aims to assess the current situation of PD in Romania by reviewing all the Romanian published studies to provide a thorough understanding of existing literature. Unlike classic systematic reviews, addressing relatively precise questions, scoping reviews offer a much larger perspective on a subject, mapping and examining emerging evidence when it is still unclear what other, more specific research questions can be posed and valuably addressed [5].

Our objective is to report data on PD epidemiology, clinical characteristics, interventions, and diagnostic challenges in Romania [2], with a large perspective on Romania’s PD research gaps [2].

## 2. Materials and Methods

The detailed protocol for the present review is published elsewhere [2]. In brief, based on the population, concept, and context (PCC) mnemonics, we performed a scoping review to answer the following research questions [6]:

- What data are published on the epidemiology of PD in Romania?
- What clinical aspects have been investigated in Romanian PD patients?
- What are the interventions introduced in Romania to reduce the burden of PD and improve the patient’s care and quality of life?
- Which are the diagnostic tests used in PD patients in Romania?

We performed a computerized bibliographic search for the following databases: MEDLINE/PubMed, EMBASE, Scopus, and Web of Sciences (up to 27 January 2021). In addition, we checked reference lists of all relevant research papers to identify possible additional studies. For the database search, we used the keywords: “Parkinson’s disease” [MeSH] AND “Romania.” These search terms were for PubMed. Searches in other data sources used similar versions of these terms, appropriate for each database. We did not apply any search filters, as we aimed to generate a broad list of studies that would be suitable for inclusion. In addition, we did not apply language restrictions.

We included all human studies reporting research on adults (over 18 years old), with at least 10 participants (P), investigating the epidemiology, clinical characteristics, interventions, or diagnostic tests in PD patients (C), conducted in Romania (C) [2].

We did not set limits on publication date, study design, or setting. We included in our scoping review published articles and also conference abstracts. Two essential factors were found to increase the probability that a study presented in an abstract will subsequently be published in full: the presence of positive or statistically significant results in the abstract and whether the authors were from an English-speaking country, who wrote their report in English. The consequence is that systematic reviews relying on fully published research may provide inaccurate or biased findings because of an over-reliance on studies with positive results or from English-speaking countries [7]. If a study was reported as a conference abstract and later in a full-text article, we did not exclude from our synthesis the abstracts. A recent systematic review assessing inconsistency between conference abstracts and full papers found that the abstract reports were frequently different from their corresponding full reports. There was a high level of inconsistency, especially concerning sample sizes, outcome measures, result presentation, interpretation, and conclusions or recommendations [8]. Nonetheless, we did not include qualitative studies because exploring barriers and facilitators for interventions was not an aim of our research. We excluded studies with individuals with secondary Parkinsonism (e.g., vascular, toxic, drug-induced, or post-infectious) or atypical Parkinsonism (e.g., corticobasal degeneration, Lewy body dementia, progressive supranuclear palsy, or multiple system atrophy). In addition, we excluded the articles where the full-text was not available and the very concise conference abstracts that did not provide data for extraction.

Two authors reviewed the title and abstract of all identified papers independently to assess the study’s eligibility. After the abstract screening, two authors independently studied the full text of the selected articles in the second stage. Disagreements were solved by a third reviewer with expertise in the domain.

To provide readers with a logical descriptive summary of the results, we charted the studies separately based on the research questions (Supplementary Materials). The results were classified under the main conceptual categories, such as: “epidemiology,” “clinical characteristics,” “interventions,” and “diagnostic tests.” Two independent reviewers extracted data. A third reviewer solved any discrepancies.

As explained in the protocol [2], we did not perform a formal assessment of the methodological quality of the included studies.

## 3. Results

**Figure 1:**
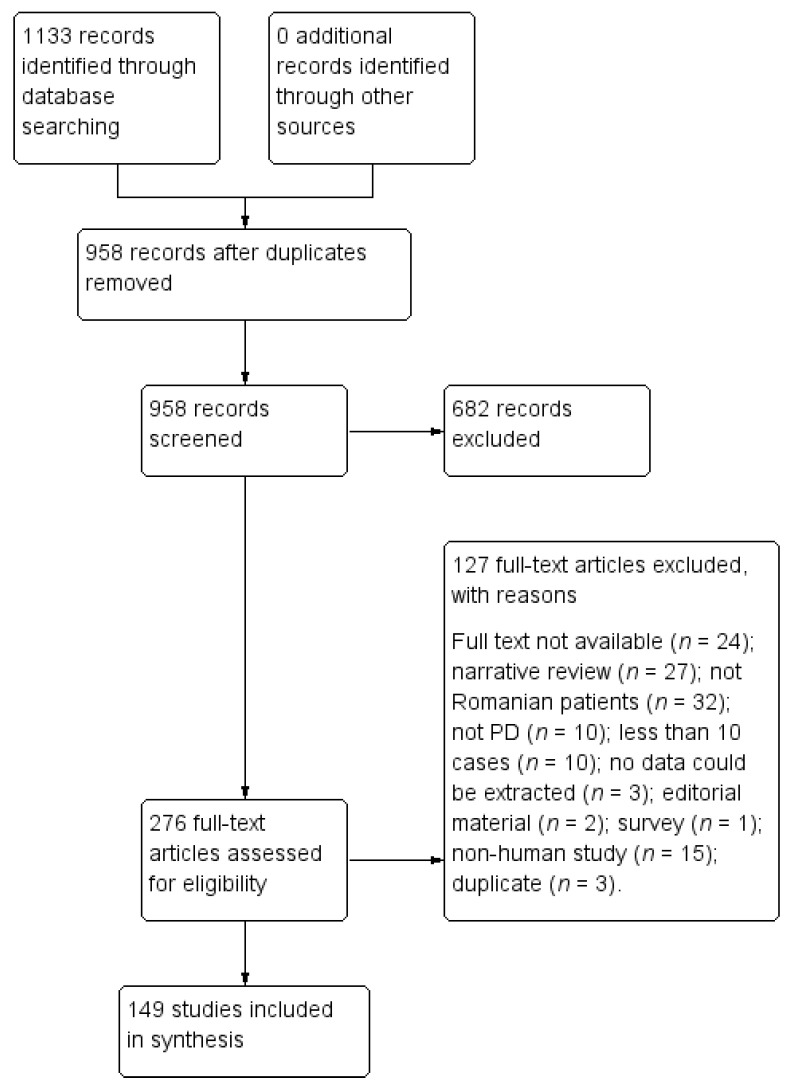
PRISMA selection flowchart.

Our search resulted in 1133 titles. We evaluated in full text 276 research papers and included in the present review 149 studies. The PRISMA diagram is presented in Figure 1.

We identified 52 studies on clinical aspects of PD [9,10,11,12,13,14,15,16,17,18,19,20,21,22,23,24,25,26,27,28,29,30,31,32,33,34,35,36,37,38,39,40,41,42,43,44,45,46,47,48,49,50,51,52,53,54,55,56,57,58,59], 35 studies investigating diagnostic tools [60,61,62,63,64,65,66,67,68,69,70,71,72,73,74,75,76,77,78,79,80,81,82,83,84,85,86,87,88,89,90,91,92,93,94], 51 studies on different pharmacological and non-pharmacological interventions [95,96,97,98,99,100,101,102,103,104,105,106,107,108,109,110,111,112,113,114,115,116,117,118,119,120,121,122,123,124,125,126,127,128,129,130,131,132,133,134,135,136,137,138,139,140,141,142,143], and only eleven epidemiological studies [1,144,145,146,147,148,149,150,151,152,153]. Some research was based in a single center, but most the university centers also participated in multicenter, international research [16,26,39,47,49,60,61,62,63,64,77,79,80,88,90,92,93,94,101,102,103,104,105,106,107,109,110,115,116,117,119,123,124,125,126,127,129,132,141,143,144,145,146].

**Figure 2:**
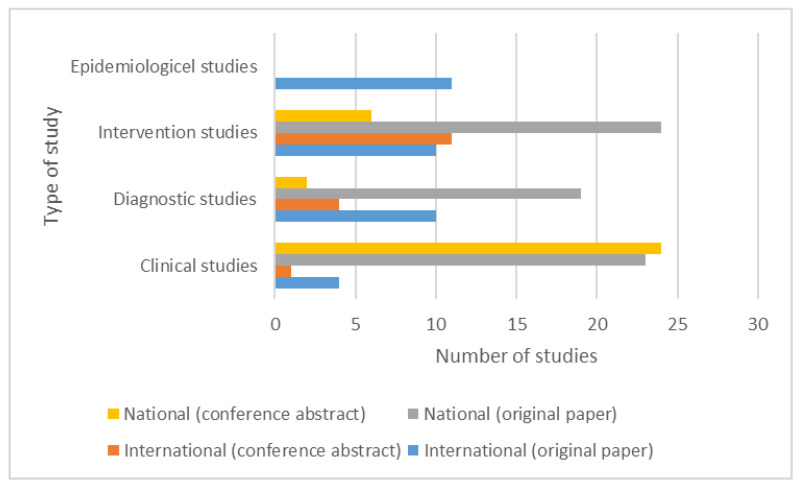
Different categories of Romanian studies.

The different categories of studies are presented in Figure 2.

**Figure 3:**
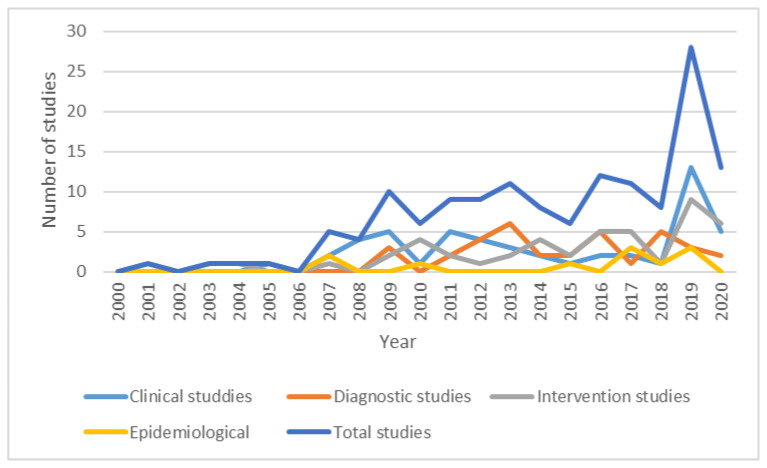
Yearly trend (number of articles) in different types of studies.

The year of publication ranged from 1973 to 2020. A considerable increase in the Romanian research on PD was observed in the latest 15 years, which largely follows international trends (Figure 3). For example, a comparative search in the PubMed database on the international publications on PD revealed that the number of articles increased each year, with a steady, global rise of research in this domain.

**Figure 4:**
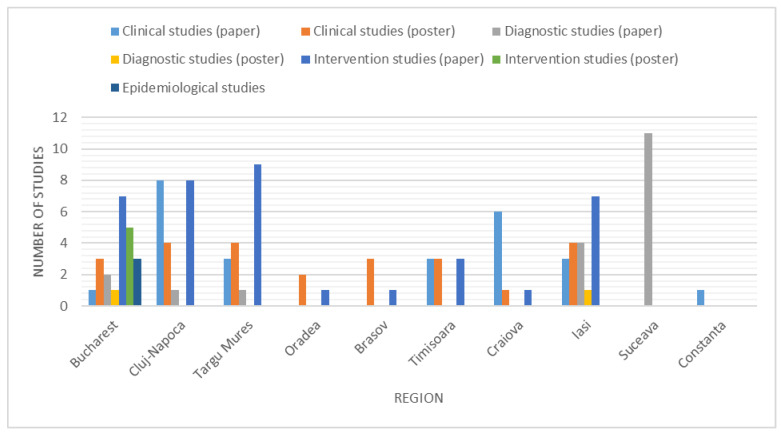
Number of studies by region.

The distribution of the different types of studies (excluding the international multicenter studies) by region is presented in Figure 4.

### 3.1. Studies Investigating Clinical Aspects of Parkinson’s Disease

**Figure 5:**
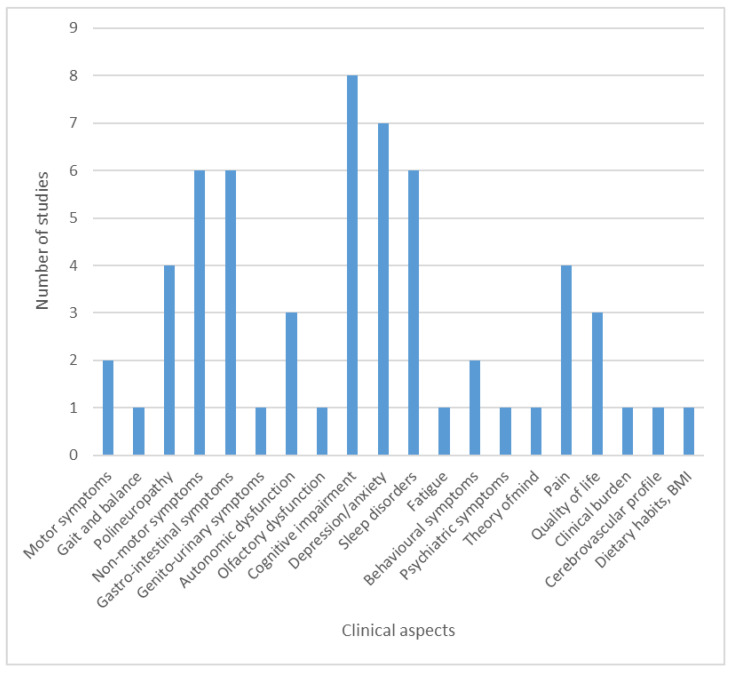
Different topics assessed by the clinical research. Some studies investigated multiple clinical aspects. Abbreviations: BMI—body mass index.

Among the 52 clinical studies (Supplemental Material: Table S1), 47 studies were done in a single national center, and 5 were multicenter, international studies [16,26,39,47,49]. Among the international studies, four were published as original papers [26,39,47,49], and one as a conference abstract [16]. The research on the clinical aspects of PD focused on a wide range of symptoms, including cognitive impairment (*n* = 8), depression and anxiety (*n* = 7), non-motor symptoms (*n* = 6), sleep disturbances (*n* = 6), pain (*n* = 4), and polyneuropathy (*n* = 4). The detailed distribution of research based on the clinical signs that were investigated is presented in Figure 5.

### 3.2. Studies on Diagnostic Tests in Parkinson’s Disease

**Figure 6:**
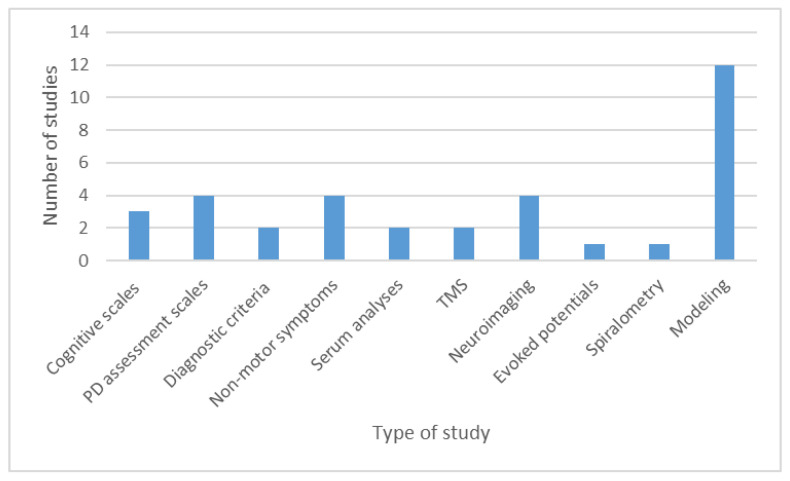
Research areas of the diagnostic studies. Abbreviations: TMS—transcranial magnetic stimulation.

Our systematic review identified 35 studies investigating different diagnostic tools (Supplemental Material: Table S2). Among them, 13 were international multicenter studies [60,61,62,63,64,77,79,80,88,90,92,93,94]; 9 were published as original papers [61,64,77,79,80,87,88,90,92], and four were abstracts of research presented at international conferences [60,62,63,93]. The rest of 41 studies were performed in national settings. A large part of the research focused on modeling studies (*n* = 12). The detailed presentation on the areas of research included in the diagnostic studies is presented in Figure 6.

### 3.3. Studies on Pharmacological and Non-Pharmacological Interventions in Parkinson’s Disease

We identified 51 intervention studies (Supplemental Material: Table S3), ranging from phase II to phase III post hoc analysis and post-marketing studies. Twenty-nine trials were national studies. In 22 studies, the Romanian participants were part of international, multicenter studies [101,102,103,104,105,106,107,109,110,115,116,117,119,123,124,125,126,127,129,132,141,143]. In the last category, 11 studies were published as original articles [101,102,115,116,117,119,124,125,129,132,141], and 11 were abstracts presented at international conferences [103,104,105,106,107,109,110,123,126,127,143].

**Figure 7:**
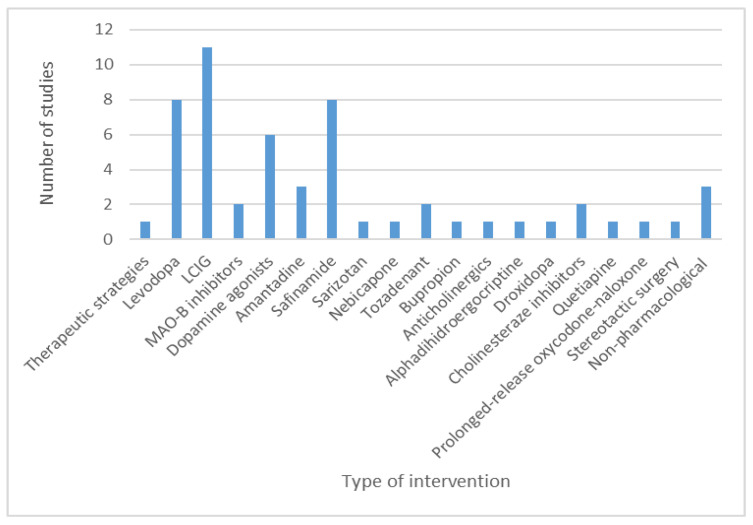
Different pharmacological and non-pharmacological interventions were investigated in Romanian Parkinson’s disease patients. Abbreviations: LCIG—levodopa-carbidopa intestinal gel; MAO-B inhibitors—monoamine oxidase type B inhibitors.

Forty-six studies investigated pharmacological interventions, one study reported on surgical procedures [95], and four studies assessed the effect of non-pharmacological interventions [112,122,130,133]. The different types of investigated interventions are presented in Figure 7.

### 3.4. Epidemiological Studies

**Figure 8:**
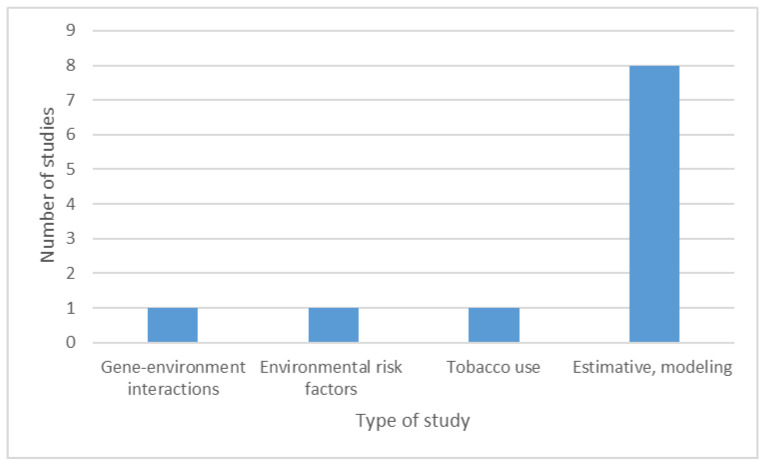
Epidemiological studies on Parkinson’s disease.

We found eleven epidemiological studies (Supplemental Material: Table S4), including one study on gene-environment interactions [145], one exploring the environmental risk factors for PD [146], one study on tobacco use [144]. In addition, we found seven international studies on the global, regional, and national burden of diseases [1,147,148,149,150,151,152,153] that also presented some data on Romania. However, in the studies on the burden of the diseases, no specific data on the Romanian PD patients were presented except in one study [1]. The data on the epidemiological studies are presented in Figure 8.

## 4. Discussion

The present scoping review provides a descriptive mapping of the literature body on PD in Romania. We identified 149 studies that assessed the different aspects of PD in Romania.

The studies on clinical aspects of PD (*n* = 52) constitute most of the research (34.89%) (see Table S1). The authors mainly investigated non-motor aspects of the disease. In addition, some studies also investigated some of the most common adverse effects of L-dopa therapy, like polyneuropathy (Table S1). One reason for the predominance of clinical studies could be that this type of observational study presents fewer technical challenges. It is less complex and less expensive than studies on diagnostic tests or interventions.

Regarding the cognitive impairment from PD, although many researchers focused on the neuropsychological aspects of the disease (Table S1), most studies used an MDS Level I assessment [9,18,39,40,45,46,138] that requires an abbreviated cognitive evaluation, either with a global scale or a limited range of neuropsychological tests [154,155]. The level II definition is based on an extensive assessment of each of the five cognitive domains (i.e., attention, working memory, executive functions, memory, visuospatial skills, and language) [154,155]. This later cognitive assessment is more expensive, time-consuming, requires highly trained personnel, and necessitates tests adapted and validated for Romanian Population. Therefore, a Level II assessment is not readily available in Romania, with implications for further research on cognitive impairment in PD.

The studies investigating different diagnostic tools for PD patients (*n* = 35) contributed to 23.48% of the research. The Romanian researchers participated in developing various clinical scales, including the non-motor symptoms scale (NMSS) [60,61], scales for outcomes in Parkinson’s disease-cognition (SCOPA-COG) [62], King’s Parkinson’s disease scale [77] and King Parkinson’s disease questionnaire [87], Parkinson’s disease composite scale (PDCS) [90,92], and the “5-2-1” screening criteria for advanced PD [93]. Nonetheless, the number of neuroimaging studies is low (*n* = 3), and there is a lack of studies on other more expensive diagnostic tools (e.g., dopamine transporters imaging, DaTscan).

From a diagnostic perspective, differential diagnoses of PD necessitate a brain MRI or, sometimes, DaTscan. Patient access to the required diagnostic tools like MRI was limited for many years due to a lack of funding. In addition, DaTscan is not available in Romania. As a result, patients with atypical Parkinsonian syndromes or genetic diseases with Parkinsonism may experience significant delays in diagnosis.

The relatively large number of modeling studies (*n* = 12) indicates a growing interest in interdisciplinary research, with possible further advancements in the field. The Romanian researchers investigated different tools for early-stage detection of PD, such as nonlinear dynamics, artificial neural networks, and neuro-fuzzy classifiers. In addition, they studied intelligent systems that could be applied to PD patients to predict the evolution of the disease over time and the possible implementation of a high-definition video system in the early management of PD (Table S2).

The studies on different interventions for PD (*n* = 51) accounted for 34.22% of publications. Most studies focused on pharmacological interventions (*n* = 46). In 22 studies, the Romanian patients were part of large, multicenter, international cohorts (Table S3). Unfortunately, none of the international studies reported any specific data for Romanian patients. Nonetheless, some researchers also focused on data solely from Romanian patients. Of note, most of the later research was retrospective, observational (Table S3).

The pharmacological studies included research on various therapeutic options, including L-dopa, dopamine agonists, and selective monoamine oxidase B inhibitors. Among the therapeutic options for advanced PD, only Levodopa–carbidopa intestinal gel (LCIG) and deep brain stimulation (DBS) are currently available in Romania. The LCIG has gained increased attention from the Romanian researchers, with the publication of 11 studies [114,118,120,125,126,132,138,139,140,142,143]. However, no studies on DBS were published in Romania, possibly due to the limited availability of the procedure. While in the western countries, there is ample knowledge and experience with managing advanced PD, in Romania, access to some therapies like apomorphine, for example, is limited; therefore, the Romanian neurologists are restricted in their management strategies when faced with a patient with advanced PD.

The number of studies on the non-pharmacological treatment of PD is limited (*n* = 4). Although globally, the research on non-pharmacological interventions is less extensive than pharmacological treatment, it has been documented to be beneficial for PD patients [156]. Therefore, developing Romanian research in this domain would be beneficial. Contextualization to the Romanian society and the low resource context, focusing on specific demographic features (i.e., multimorbidity), activity limitations, and participation restrictions, may increase Romanian PD patients’ quality of management and care.

The present systematic review revealed only eleven epidemiological studies (7.38%), with three studies focusing on genetic and environmental factors [144,145,146]. The rest of the eight papers communicate research on the global burden of diseases and are based on statistical modeling. To date, data on the number of Romanian PD patients are lacking, with only some estimative numbers. In addition, there is no study on genetic forms of PD.

Internationally, there is growing evidence of the benefits of precision medicine in PD. A personalized approach, tailoring PD treatments based on the patient’s individual genotype, may help reach disease modification [157]. Clinical trials targeting genetic forms of PD, like GBA-associated and LRRK2-associated PD, are timely and of great interest.

Therefore, well-designed large-scale epidemiological, genetic studies are much needed in Romania. In addition, a potential future action could be the initiation of a national registry of PD patients. Nonetheless, the implementation of such a registry may prove to be challenging. For example, such a registry may include non-PD Parkinsonism cases due to a lack of movement disorders specialists and potential PD overdiagnosis [158].

The present scoping review identified several gaps in research in PD research in Romania, providing details on key implications for research and further need for primary studies [6,159]. These gaps could be partially explained by the relatively reduced number of neurologists specializing in movement disorders. For example, in Romania, there are 12 medical universities; however, we found publications only from 10 centers. In addition, there is a limited number of PD nurses and occupational therapy specialists. Therefore, training young specialists with a special interest in movement disorders would be of many benefits.

Other identified gaps comprise limited access to some treatments (e.g., apomorphine infusion, DBS) or some diagnostic investigations (e.g., DaTscan). In addition, increasing the involvement of healthcare policymakers and public awareness on PD would enable further research in movement disorders in this Eastern European country. Specialist medical training on movement disorders, educational activities, research methodology, and support for grant writing initiatives would also help to increase the PD research and care in Romania.

The main identified gaps, possible causes for these gaps, and suggested actions are presented in Supplemental Material Table S5. Therefore, the present scoping review provides implications for research and indicates areas of primary research where future studies are needed. In addition, we provide a basis for further qualitative studies, exploring barriers and facilitators for different actions and interventions that can contribute to a comprehensive understanding of people’s values, attitudes, and beliefs across PD patient populations and healthcare contexts to inform patient-centered practice and policy [160].

## 5. Conclusions

To our knowledge, this is the first scoping review dedicated to the PD literature in Romania. Overall, we found a steady increase in the number of published studies, reflecting a positive change in the PD research in this region. We provided a complete picture of the current state of knowledge, research, and practices. Furthermore, we identified several gaps in this area. Therefore, a joint effort of local neurologists, healthcare providers, public health specialists, policymakers, and international PD and movement disorders organizations will help overcome the current challenges and provide better research, management, and care of PD patients from Romania.

## Supplementary Materials

The following are available online at https://www.mdpi.com/article/10.3390/brainsci11060709/s1, Table S1: Clinical studies; Table S2: Diagnostic test accuracy studies; Table S3: Intervention Studies; Table S4: Epidemiological studies. doi:10.6084/m9.figshare.14501469.
