# Supplementary material for: Parkinson’s Disease in Romania: A Scoping Review
Source: Brain Sci. 2021 May 27;11(6):709. doi: 10.3390/brainsci11060709 (PMC8226866; doi:10.3390/brainsci11060709)
Supplement: Supplementary file 1 [file brainsci-11-00709-s001.zip › brainsci-1221717-supplementary.pdf]

Table S1. Clinical studies.

Study	Town / region	Topic	Objective	Study type	Methods	Main results
Szocs 2005 (poster)	Tirgu-Mures	Cognitive impairment	To investigate the cognitive functions in PD	N/R	31 PD patients<br>Beck Depression Scale, Rey complex figure (visual<br>memory), Ranschburg - Ziehen test (short-term memory), MMSE	21 patients with motor fluctuations, 10 patients without motor fluctuations<br>The patients presenting motor fluctuations suffer of depression – including in 'on' stage – in a higher degree (although not significantly higher) than patients with stabile motor functions. There was no significant difference concerning mental functions between the two groups.
Popescu 2007 (poster)	Bucharest	Quality of life	To explore the statistical correlation between the PDQ<br>39 questionnaire results and 'on' time results from the patient's diaries.	N/R	11 Idiopathic PD with motor fluctuations.<br>Exclusion criteria were any other serious diseases that could alter quality of life.<br>Assessment: UPDRS, H&Y, Schwab and England ratings, PDQ 39	Motor fluctuations started from 0.08 to 8 years before inclusion, and dyskinesias were present in 7 of 11 patients.<br>UPDRS 'off' scores: 44-106; Schwab and England ratings: 50% - 80% in 'off'; H&Y staging: 2-4 (in 'off' state)<br>The PDQ 39 scores were correlated with on-time in patient diaries, and inversely correlated with off-time in diaries and Schwab and England rating.<br>No correlation tendency was noted between PDQ 39 score and age of the patient, duration from disease onset, and presence of dyskinesia.
Reisz 2007 (poster)	Timisoara	Depression	To describe the depressive-like neuropsychiatric<br>complaints in PD patients	N/R	48 PD patients with MMSE >26<br>Assessment: duration of disease, the stage of disease, UPDRS, Beck Depression Inventory, HAM-D, HAM-A, Geriatric Depressive, a list of 50 depressive-like and depressive items, and a quality-of-life questionnaire.	The actual scale used for identifying depression tends to overestimate the incidence of the depression in PD because of an overlapping between symptoms of PD and some symptoms of depressive disorder (flat affect, inability to work, fatigue, preoccupation with ill health, loss<br>of desire, and reduction in libido).<br>The emerged list with depression-like symptoms could be a list of those depressive aspects mediated by dopaminergic systems. The list affords us to characterize this particular group of patients and we could compare neuropsychiatric traits in Parkinson's disease with similar data in Europe
Mihancea 2008 (poster)	Oradea	Non-motor symptoms	To evaluate the non-motor symptoms of PD	N/R	110 PD patients	55% of the patients presented non-motor symptoms, occurring at the beginning of the disease, during its evolution and even before the onset of the non-motor symptoms.<br>The non-motor symptoms found in our patients were (according to their frequency): cognitive disorders, constipation, sleep disorders, depression, dementia, hallucinations, olfactory disorders, muscle cramps, fatigue, joint and muscle pains, numbness, brady-psychasthenia, akathisia, orthostatic hypotension, gastrointestinal disorders, anxiety, panic attacks, irritability, bladder disorders, erectile dysfunctions, tachycardia, cold or hot sensation, profuse

						sweating, seborrheic dermatitis
Reisz 2008 (poster)	Timisoara	Anxiety, depression, sleep disorders	To perform a quantitative and qualitative analysis of the most frequent psycho-pathological symptoms present in PD	N/R	63 PD patients<br>Assessment: BDI, HAM-A, HAM-D, GDS, HADS), Epwort Sleepiness Scale, Parkinson's Disease Sleep Scale and a questionnaire on the duration of sleep and the subjective perception of the quality of sleep.	31.7% of patients presented signs of medium and major depression. Anxiety symptoms was present in 88.9% of cases, and was of medium intensity. The consciously lived anxiety disorder was much rarer (14.3%).<br>84.1% of patients presented sleep disorders. Nevertheless, only 58.7% had a perceived suffering. The rest had no major complaints related to the sleep disorder, even if they mentioned short sleep periods and frequent nightly awakenings.
Stoian 2008 (poster)	Targu Mures	Depression	To assess the presence of the depressive syndrome in PD patients	N/R	92 PD patients<br>Assessment: H&Y scale, Beck Depression scale	Authors found significant age-related differences regarding the occurrence of the depressive syndrome: 34.69% of the patients between 50–70 years; 18.42% of the patients between 70–90 years; 16.66%of the patients between 80–90 years. Patients with postural instability presented a higher incidence of depression.<br>Authors did not find a statistically significant correlation between the incidence of depression and the H&Y degree of motor activity impairment, but the incidence of depression was directly correlated with postural instability and cognitive deterioration.
Szocs 2008 (poster)	Targu Mures	Quality of life	To investigate the impact of motor fluctuations, depressive symptoms and sleep disorders on the quality of life of PD patients	N/R	36 PD patients<br>Assessment: UPDRS, Parkinson's Disease Quality of Life – 39, Hamilton Depression Scale, Epworth Sleepiness Scale, and information concerning personal data, duration of disease, duration and patterns of motor fluctuations	QoL was influenced significantly by duration of motor fluctuations, (the longer the daily off-time, the worse the QoL), and also by severity of motor fluctuations, though not significantly.<br>Daytime sleepiness and depression scores did not show any correlation with QoL, but both showed a positive correlation with the disease duration, Hamilton scores also were significantly increased with duration of motor fluctuations.<br>Higher Hamilton scores were associated with higher Epworth Sleepiness scores.<br>Motor fluctuations worsen the quality of life of PD patients, aggravating the depressive symptoms.<br>There was no correlation between daytime sleepiness, depression scores and quality of life.
Martinez-Martin 2009 (poster)	Multicenter, Brasov	Non-motor symptoms	To detect gender differences in NMS using the Non-Motor Symptoms Scale (NMSS)	Cross-sectional	750 PD patients<br>Assessment: NMSS, H&Y staging, motor examination and motor complications scales.	58.5% of patients were men.<br>No significant differences were observed in age, age at onset, duration of disease, H&Y staging, and motor manifestations and complications between men and women. NMSS total score was 54.01±41.39 for men and 60.76±46.34 for women (p = 0.09). Sleep/fatigue, Mood/apathy, and Miscellaneous domains showed significantly higher scores for women whereas men showed higher scores for Sexual function domain. Regarding NMSS items, women had significant more problems than men with falls because of fainting, more difficulties falling or staying asleep, more anxiousness, and anhedonia. On the contrary, men showed more troubles with drooling and problems

						having sex.
Muntean 2009 (poster)	Cluj-Napoca	Pain	To investigate the influence of pain on the quality of life of patients with PD	N/R	50 PD patients<br>Assessment: Visual Analogue Scale (VAS) and the<br>Brief Pain Inventory (Romanian version),<br>Montgomery Asberg Depression Scale (MADRS),<br>Parkinson's Disease Questionnaire (PDQ-39).	60% of patients reported chronic pain. The most frequent concomitant pathologies as a possible cause of pain<br>were osteoarticular and cardiovascular diseases. However, 44% of the patients considered pain to be caused by PD. The average intensity of pain on VAS was 6 on a scale from 0 (no pain) to 10 (worst pain). The intensity of pain correlated with higher scores on PDQ-39. Patients who experienced pain had more severe depressive symptoms. There was no correlation between pain severity and disease staging.
Pirscoveanu 2009	Craiova, Timisoara	Cognitive impairment	To assess the cognitive status in PD patients	Case-control	58 PD patients (H&Y = 1), 62 normal controls<br>Assessment: MMSE and the revisited Adenbrooke's Cognitive Examination (ACE-R) at baseline, 6 months, 1 year	Baseline: PD patients presented MMSE (mean) = 27.6; ACE-R (mean) = 89.3; controls presented MMSE (mean) = 28.7; ACE-R (mean) = 90.1<br>After 1 year: PD patients presented MMSE (mean) = 25.1; ACE-R (mean) = 84.4; controls presented MMSE (mean) = 26.8; ACE-R (mean) = 88.2<br>Patients with H&Y 2 had greater cognitive impairment compared with H&Y = 1<br>Cognitive impairment was found across every ACE-R cognitive domain
Sandulescu 2009	Craiova	Gait and balance	To estimate the correlation<br>between motor performances and balance and gait disorders in patients with PD.	N/R	36 PD patients, H&Y stages 2 -4.<br>Assessment: UPDRS part 3 and 4, Tinetti Assessment Tool: Balance and Gait (TABG) and The Dynamic Gait Index (DGI).	The lower scores on both TABG and DGI were strongly correlated with stage 3 and 4 of the disease, scores higher than 27 points on UPDRS part 3, hypokinesia, rigidity, freezing of gate and "off" periods.<br>There was no correlation with age or resting tremor.
Tudorica 2009	Craiova	Anxiety	To evaluate anxiety in PD patients	N/R	37 non-demented PD patients (H&Y = 1-2)<br>Assessment: Zung Self-Rating Anxiety Scale, Hamilton Anxiety Rating Scale, Hamilton Depression Rating Scale	32.4% of patients presented anxiety. An association between anxiety and depression was found in 48.3% of patients.<br>Anxiety was more frequent in patients left-side motor symptoms.
Capusan 2011 a	Cluj-Napoca	Cognitive impairment	To evaluate the presence of cognitive impairments in early stages of PD, with focus on executive function and attention deficits.	Case-control	30 patients diagnosed with PD (stage I and II) and 30 controls.<br>Exclusion criteria MMSE < 27.<br>Every participant was evaluated with the following tests: Go-No-Go, Word List Memory, Spatial Target Detection Test and PEAT, all from COGTEST program.	The PD patients presented deficits of executive functions, impaired working memory and social cognition, with prolongation of reaction time at test completion.
Georgescu 2011 (poster)	Timisoara	Gastrointestinal symptoms	To evaluate dyspepsia in PD patients by assessment of gastric motility in order to improve treatment and patient's quality of life.	N/R	27 PD patients treated with levodopa or dopamine agonists, with associated nausea and vomiting<br>Investigation: ultrasound assessment of gastric motility (Bolondi method).<br>Patients received a treatment with Trime-	Before therapy 55.56% of patients presented delayed gastric emptying, 25.92% had normal motility and 18.51% presented rapid emptying<br>Symptom's severity scores before therapy were: mild (29,52%), medium (51.85%), or severe (18.51%).<br>severe.

					butine 300 mg/day for 3 months.<br>After the treatment authors repeated the ultrasound<br>exam of gastric motility.	Many patients presented gastric motility disorders (74.07%), most of them having delayed emptying.<br>There was a a satisfactory response to the treatment with Trimebutine with improvement of symptoms severity<br>index and gastric motility, more expressed in patients with delayed gastric emptying.
Capusan 2011 b	Cluj-Napoca	Cognitive impairment	To evaluate the presence of cognitive impairments in early stages of PD<br>To investigate if there was a link between the cognitive impairment and clinical and imagistic data.	N/R	30 patients diagnosed with PD (stage I and II)<br>Cognitive evaluation: COGTEST software.	The authors concluded that, even if cognitive impairment was detected in patients with tremor, significant deficits of attention, executive function and working memory were found in subjects with excessive rigidity.
Muntean 2011 (poster)	Cluj-Napoca	Sleep disturbances	To assess nighttime sleep in PD patients using bedside questionnaires and to analyze their impact on different aspects of the quality of life.	Prospective, cohort	122 consecutive PD patients<br>Assessment: Parkinson's Disease Sleep Scale (PDSS), Parkinson's Disease Questionnaire (PDQ-39).	The mean disease severity was 2.83 on the H&Y scale. Night-time sleep was impaired in 43% of patients, considering a total PDSS score of less than 90 as<br>abnormal.<br>The most frequent reported night-time problems were muscular cramps, involuntary limb movements, pain<br>and fragmentation of sleep with frequent awakenings, with<br>a major impact on the quality of life.
Tudorica 2011 (poster)	Craiova	Pain	To estimate which factors correlate with pain in PD patients.	N/R	85 PD patients<br>H&Y scale, UPDRS-3 and 4, MMSE, 5-Point Verbal Rating Scale, 6-Point Behavioural Rating Scale and 11-Point Box Scale.<br>The patients were divided in 2 groups according to the presence of pain (group A) or its absence (group B).	35 patients (41.1%) composed group A. The pain<br>was localized mostly on back (57.1%) and its intensity<br>varied from mild (54.2% of cases) to severe (11.4%). There were no statistically differences between groups regarding gender, length of the disease and UPDRS score.<br>Pain correlated with younger age, "off" state, dystonia and low dose of L-Dopa.
Martinez-Martin 2012	Multicenter, Brasov	Non-motor symptoms	To evaluate the prevalence and severity of non-motor symptoms (NMS) by gender in an international sample of PD	Exploration of a data set from an international, cross-sectional, observational study, and the baseline data from a longitudinal study	951 PD patients (14 countries)<br>Assessment: Non-motor Symptoms Scale (NMSS).	No specific data on Romanian patients.<br>The most prevalent symptoms were nocturia (64.88%) and Fatigue (62.78%). The most prevalent affected domains were sleep/fatigue (84.02%) and miscellaneous (82.44%). Fatigue, feelings of nervousness, feelings of sadness, constipation, restless legs, and pain were more common and severe in women.<br>Daytime sleepiness, dribbling saliva, interest in sex, and problems having sex were more prevalent and severe in men.<br>Regarding the NMSS domains, Mood/Apathy and Miscellaneous problems (pain, loss of taste or smell, weight change, and excessive sweating) were predominantly affected in women and Sexual dysfunction in men. No other significant differences by gender were observed.

Muntean 2012 (poster)	Cluj-Napoca	Non-motor symptoms	To assess the impact of pain, night-time sleep disturbances and depression, as non-motor symptoms, on the quality of life of PD patients.	N/R	122 PD patients (H&Y = 1-4)<br>Assessment: Parkinson's disease Questionnaire (PDQ-39), Brief Pain Inventory, Parkinson's disease Sleep Scale (PDSS), and Montgomery Asberg Depression Rating Scale (MADRS).	The QoL of PD patients who reported pain was more severely impaired when compared with PD patients without pain.<br>Night-time sleep disturbances had an important role in altering QoL in PD patients.<br>Depression was another decisive factor for the QoL
Susin 2012	Cluj-Napoca	Activities of daily living and quality of life	To describe the life of patients with PD in the light of daily activities, general<br>levels of cohabitation and well-being		16 PD outpatients<br>Assessment: age, gender, age at disease onset, marital and living status, working status, information related to H&Y stage, and side affected PDQ-39, Instrumental Activities of Daily Living (IADL), MMSE; other diagnoses and treatment status were taken from medical files. In-person interview.	Women were more affected compared with men regarding ADL and IADL.<br>Patients were emotionally affected in all stages, often only for a limited period and secondarily to adverse psychosocial circumstances such as stress and<br>loss, or to disappointment for failing to live up to one's own expectations.<br>Quality of life started to be affected at early in the disease, initially due to the motor disturbances which caused impairments in daily activities or negative feelings.<br>Patients with PD stage 1 or 2 had a better quality of life, a better capacity to perform in daily activities compared<br>with those in the final stages. But, at any stage, a rapid deterioration, or the development of treatment complications, or the worsening symptoms caused a lower quality of life.
Tohanean 2012	Cluj-napoca	Olfactory dysfunction	To evaluate the olfactory dysfunctionin recently diagnosed PD patients	Retrospective, descriptive	40 PD patients<br>Assessment: 12-marker Smell Test: Sniffin' Sticks Screening 12	87.5% of PD patients presented hyposmia or anosmia<br>There was no correlation between olfactory dysfunction and the duration or the stage of PD
Dumitru 2013 a (poster)	Iasi	Sleep	To examine the prevalence of sleep problems in PD patients in Romania, and their associated factors	N/R	44 consecutive PD inpatients were included in a study of non-motor symptoms, including sleep problems.<br>Assessment: Parkinson's Disease Sleep Scale (PDSS). Factors associated with sleep were also investigated, with special emphasis on severity of PD, fatigue, mental health and restless legs syndrome (RLS).	The mean age was 67.8 years (range 35-74); the mean H&Y stage was 2.13 (SD 0.89), and the mean UPDRS part III was 22.6 (SD 11.5).<br>Sleep problems were common among PD patients. While only 17% of the sample had an overall score below 82 on the PDSS, 70% of the patients had a score below 5 on one item.
Dumitru 2013 b (poster)	Iasi	Depression and apathy	To establish the prevalence and demographic and clinical correlates of depression and apathy in a sample of PD inpatients.	N/R	44 inpatients with PD<br>Assessment: Starkstein's Apathy Scale (AS), the<br>17-item Hamilton Depression Rating Scale (HDRS-17), H&Y staging, UPDRS and MMSE.	Apathy coexisted with depression in 34.1% of PD patients, compared with depression without apathy in 4.6%, and<br>apathy without depression in 22.7%.<br>Apathy was associated with higher UPDRS score, lower MMSE score and earlier HY stages.<br>Depression was correlated with more advanced HY stages and younger age of patients with PD
Dumitru 2013 c (poster)	Iasi	Depression and apathy	To examine the prevalence of depression and apathy in a hospitalized	Cross-sectional	40 inpatients with PD<br>Assessment: Starkstein Apathy scale (AS), Hamilton Depression Rating scale	Patients were classified into four groups: (1) patients with apathy and depression 37.5%, (2) apathy without depression 22.5%, (3) depression without apathy 5%, and (4) neither depression, nor

			population with PD.<br>To examine the demographic and clinical correlates of depression and apathy and to assess whether apathy can be present as a primary behavioral disorder.		(HAMD), MMSE, H&Y scale, and UPDRS.	apathy 35%.<br>Depression was associated with more advanced H&Y stages and younger age of patients, while apathy was correlated with higher UPDRS score, lower MMSE score and higher L-dopa dosage.
Perju 2014	Cluj-Napoca	Cerebrovascular profile	To assess the cardiovascular and cerebrovascular profiles of PD patients.	Cohort	The cardiovascular risk factors of 126 PD patients were assessed according to laboratory tests (fasting blood sugar, serum cholesterol, triglycerides, and total lipids), Doppler ultrasound examinations and personal histories of cerebrovascular disease (ischemic/hemorrhagic), cardiovascular disease (myocardial infarct or angina confirmed by electrocardiogram), hypertension and diabetes.<br>All patients underwent cerebral structural imaging procedures: brain Ct or MRI	58.73% of the patients presented with hypertension, with a slight predominance of female patients (65.38% vs 47.92%).<br>Carotid or vertebral atheromatosis was present in 30.95% and 22.22% of patients, respectively, and was statistically correlated with the presence of ischemic lesions on cerebral imaging.<br>Regarding the CT findings, 28.21% of patients presented with cortical atrophy that was not correlated with any of the investigated cardiovascular factors.
Vasile 2014	Bucharest	Non-motor symptoms	To evaluate the prevalence of non-motor symptoms in PD	Prospective, observational	70 PD patients<br>Assessment: NMS-QUEST	Authors found the following prevalence data: recent memory disturbances (65%), concentration problems (45%), sad mood or depression (57%), anxiety (40%), sleep disturbances (80%), symptoms possibly due to orthostatic hypotension (approx. 70%), urinary problems (80%), bowel disorders (approx. 60%), drooling (approx. 50%), hyposmia or ageusia (approx. 40%)
Baetu 2015 (poster)	Bucharest	Polyneuropathy	To determine the etiologic factors of polyneuropathy in patients with advanced PD, who received LCIG treatment	N/R	28 patients with advanced PD in treatment with LCIG were examined by EMG and serum level of B12 before treatment and 6 and 12 months after treatment.	The authors found some cases of severe sensorimotor polyneuropathy with both subacute and chronic onsets, rarely associated with vitamin B12 imbalance. No severe changes in vitamin B12 level were found. This may reflect a rare complication or a side effect of LCIG.
Georgescu 2016	Timisoara	Gastrointestinal disorders	To investigate some lower gastrointestinal non-motor symptoms in a group of patients with PD.	N/R	40 PD patients, with levodopa or dopamine agonist treatment, were randomly selected.<br>In the non-motor symptoms questionnaire (NMS-Quest), regarding GI complaints, the following were recorded: abdominal pain, bloating, and constipation of mild-to-moderate severity.<br>Laboratory studies, abdominal ultrasound, and upper and lower digestive endoscopies	20-25% of the patients had more than one autonomic complaint, 15-25% had more than two complaints, and 10% had more than three complaints.<br>Nausea and dysphagia were very frequently associated with other non-motor features.<br>About 75% of patients from the first group and 70% of patients from the second group had nausea. Dysphagia was recorded in 50% of the patients from the first group and in 40% of the patients from the second group.

					were performed to rule out organic issues.<br>Treatment (3 months): 20 patients received trimebutine 200 mg three times daily half an hour before meals. The other 20 patients received probiotics (60 mg per-tablet of two lactic bacteria: Lactobacillus acidophilus and Bifidobacterium infantis), 2 times/day, 1 hour after meals	Other symptoms such as urinary urgency, nocturia, orthostatic hypotension, and excessive sweating were present in less than 35% of all the patients.<br>Treatment with probiotics could improve abdominal pain and bloating as much as with trimebutine, but less for constipation with incomplete evacuation, where trimebutine showed better results
Jurcau 2016 (poster)	Oradea	Autonomic dysfunctions and sleep disturbances	To assess potential clinical risk factors for dementia in PD	Prospective, cohort	81 PD patients<br>Assessment: H&Y staging, UPDRS, Unified Multiple System Atrophy Rating Scale (UMSAR), Insomnia Severity Index (ISI), MMSE, 10-item Neuropsychiatric Inventory (NPI) and Hamilton Depression Rating Scale (HAMD); blood pressure was measured supine and after 2 minutes of standing (at inclusion and at 2-year follow-up).	The MMSE score at 2-year follow-up was influenced by the ISI scores and the presence of autonomic dysfunctions.<br>Neither baseline dose of levodopa, UPDRS score nor HAMD score predicted development of dementia.<br>In non-demented patients the MMSE score at follow-up was influenced by the dose of levodopa, and signs of autonomic dysfunction.
Diaconu 2017 (poster)	Brasov	Fatigue	To evaluate the severity of fatigue in PD patients, as well as possible risk factors and the impact on quality of life	Prospective, cohort	52 patients with PD.<br>Assessment: Fatigue Symptom Inventory (FSI).	For the first part of the scale (questions 1-4), most of the patients rated a moderate level of fatigue (5 out of 10 points).<br>Regarding the perceived interference with quality of life, most of the patients rated a mild-to moderate impact of fatigue (mean of 3.6 out of 10 points for males). Most of the patients (36.3% males, 27.2% females) felt fatigued during 3 out of 7 days.<br>The patients felt the fatigue mostly during evening. Levodopa equivalent dose and depression were independent risk factors for presence and degree of fatigue.
Kramberger 2017	Multicenter, Brasov	Cognitive impairment	To describe the rate and clinical predictors of cognitive decline in dementia with Lewy bodies (DLB), and compare the findings with Alzheimer's disease (AD) and Parkinson's disease dementia (PDD) patients.	Longitudinal, cross-sectional	1,290 patients (835 DLB, 198 PDD, and 257 AD) were available from 18 centers<br>No specific data on Romanian patients.<br>Assessment: MMSE with up to three years longitudinal data.	The mean annual decline in MMSE score was 2.1 points in DLB, compared to 1.6 in AD ($p = 0.07$ compared to DLB) and 1.8 in PDD ($p = 0.19$). Rates of decline were significantly higher in DLB compared to AD and PDD when baseline MMSE score was included as a covariate, and when only those DLB patients with an abnormal dopamine transporter SPECT scan were included. Decline was not predicted by sex, baseline MMSE score, or presence of specific DLB core features.
Davidescu 2018 (poster)	Bucharest	Neurocognitive and mood disorders	To assess neurocognitive impairments and mood disorders in PD	N/R	Patients were assessed with Beck Depression Inventory and Hamilton Anxiety Rating Scale, MMSE and Sunderland clock test<br>Caregivers were assessed with Neuropsychiatric Inventory for checking patients'	45.45% of patients had a depressive disorder, 23.38% had anxious troubles and 22.08% of them had a mixed state.<br>24.68% presented a mild cognitive impairment and 11.69% with an associated major neurocognitive disorder according DSM 5.

					behavioral problems	
Tohanean 2018	Cluj-Napoca	Psychiatric symptoms	To assess the non-motor symptoms in early stages of PD		43 PD patients<br>Assessment: NMS-PD QUEST	Approximately 1/3 of patients complained about 5 symptoms simultaneously. The most common symptoms reported were depression (81.39%), impaired attention (79.06%), memory problems (74.41%), and complaints related to impaired sleep.
Criciotoiu 2019 a	Craiova	Motor symptoms	To evaluate the necessity of LCIG depending the PD motor subtypes and age at onset of the disease	N/R	70 patients with PD (36 with oral levodopa, 34 with LCIG)<br>The UPDRS assessment was performed in on state. The patients were classified as tremor-dominant type (TDT), akinetic-rigid type (ART) and mixed type (MT).	Authors found a statistically significant correlation between the age at onset of PD and the motor subtype of the disease.<br>The lower the age at diagnosis, the more the patients have reached the need for LCIG.<br>Regarding the motor subtype, the study found that the mixed typed request more frequently LCGI.
Criciotoiu 2019 b	Craiova	Non-motor and motor symptoms	To evaluate the correlation between motor and non-motor symptoms in PD patients	N/R	72 patients with PD.<br>Each patient was evaluated using Non-motor Symptoms Questionnaire for Parkinson Disease. The motor status was assessed with UPDRS part 3 (motor part)	Authors found a correlation between the non-motor state and the motor symptoms but not in all non-motor domains.<br>The digestive symptoms, the, cardiovascular, sleep and miscellaneous correlated with the motor symptoms but urinary symptoms, memory, hallucinations, depression and sexual dysfunction did not show an interdependence with the motor state.
Criciotoiu 2019 c	Craiova	Gastrointestinal symptoms	To assess the impact of digestive dysfunctions on quality of life in PD	N/R	60 patients with PD (H&Y = 2-5)<br>Exclusion: <8 years of education and at MMSE <l 25 points, history or evidence of other medical condition that could interfere with quality of life<br>The digestive dysfunction was evaluated using the<br>digestive domain of self-assessment Non-motor<br>Symptoms Questionnaire for Parkinson's Disease (NMSQ)	Authors found a low correlation between digestive dysfunctions and the severity of the disease, but a statistically significant correlation between quality of life and the stage of the disease.<br>There was a statistically significant correlation between digestive dysfunctions and quality of life.
Cuciureanu 2019 a	Iasi	Cognitive and behavioral symptoms	To investigate cognitive and behavioral changes in PD patients	Observational, prospective	112 patients with PD<br>Assessment: UPDRS, H&Y scale, MoCA, Hamilton Depression Scale (HAM-D), Questionnaire for Impulsive-Compulsive Disorders in PD, Global Assessment of Functioning scale	The longer the duration of the disease, the more critical impulse-control disorders were, especially shopping, lobbyism, and punding. Hypersexual behavior appeared to be age and gender-dependent. Depression was interconnected with impulse-control disorders: subjects with severe depression had more shopping compulsions. Antiparkinsonian treatment influenced the MoCA score. Subjects treated only with levodopa had lower scores at temporal and spatial orientation testing. Dopamine agonists use was associated with less cognitive dysfunctions.
Cuciureanu 2019 b (poster)	Iasi	Cognitive and behavioral symptoms	To evaluate the psychological and psychiatric disturbances in PD	Observational, prospective	94 patients with PD<br>Assessment: UPDRS, H&Y scale, MoCA, Hamilton Depression Scale (HAM-D), Questionnaire for Impulsive-Compulsive Disorders in PD, Global Assessment of	60% of patients presented cognitive impairment and 84% depression. 23% of them had compulsive impulsive behavior (punding, >sexual).<br>The level of education, gender, age, duration of the illness, severity, the treatment was found to influence the type and evolution of

					Functioning scale (GAF)	psychiatric manifestations.<br>There was a correlation between depression and duration of illness. The cognitive performances were correlated with age and treatment. The compulsive-impulsive<br>manifestation was corelated with age and duration of illness.
Fasano 2019	Multicenter (18 countries)	Clinical burden of advanced Parkinson's disease (APD)	To determine the proportion of APD vs. non-advanced PD (non-APD) patients attending specialist PD clinics and to demonstrate the clinical burden of APD.	Observational, cross-sectional	2615 PD patients at 128 movement disorder centers in 18 countries (95 APD, 66 non-APD patients)<br>Motor and non-motor symptoms, activities of daily living, and quality-of-life end points were assessed. The correlation between physician's global assessment of advanced PD and the advanced PD criteria from a consensus of an international group of experts (Delphi criteria for APD) were evaluated.	No specific data for Romanian patients.<br>According to physician's judgment, 51% of patients were considered to have APD. There was a moderate<br>correlation between physician's judgment and Delphi criteria for APD.<br>Activities of daily living, motor symptom severity, dyskinesia duration/disability, "Off" time duration, non-motor symptoms, and quality-of-life scores were worse among APD vs. non-APD patients.<br>APD patients (assessed by physicians) had higher disease burden by motor and non-motor symptoms compared with non-APD patients and a negative impact on activities of daily living and quality of life.
Irene 2019	Constanta	Genitourinary dysfunction	To estimate the prevalence of patient-reported genitourinary dysfunction symptoms in PD	N/R	86 patients with idiopathic PD<br>Assessment: Scale for Outcomes in Parkinson's Disease for Autonomic Symptoms (SCOPA-AUT) as a self-administered questionnaire	98.8% of patients reported at least one genitourinary dysfunction. The most common urinary symptom was nocturia (95.3%), followed by pollakiuria (82.5%), difficulty passing urine and urge to urinate (76.7%), incomplete bladder emptying (75.5%), urinary incontinence (67.4%). Most of the study subjects experienced genitourinary symptoms only "sometimes". None of the investigated PD patients affected by genitourinary symptoms used specific medication therapy.
Martinez-Martin 2019	Multicenter, Brasov	Nocturnal sleep dysfunction, pain	To explore the influence of the various pain subtypes experienced by PD patients on sleep.	Observational, cross-sectional	300 PD patients (1 centers across UK, 1 center from Romania)<br>Assessment: PD Sleep Scale-Version 2 (PDSS-2), King's PD Pain Scale (KPPS), King's PD Pain Questionnaire (KPPQ), Visual Analog Scales for Pain (VAS-Pain), and Hospital Anxiety and Depression Scale.	No specific data for Romanian patients.<br>According to the PDSS-2, 99.3% of the sample suffered from at least one sleep issue. Those who reported experiencing any modality of pain suffered significantly more from sleep disorders than those who did not. The PDSS-2 showed moderate-to-high correlations with the KPPS, KPPQ, and VAS-Pain.<br>The KPPS and KPPQ were the most relevant predictors of sleep disorders (as per the PDSS-2), although<br>following exclusion of PDSS-2 pain items, depression was the relevant predictor. Depression and anxiety were<br>the most relevant predictors in the analysis involving the VAS-Pain.<br>Pain showed a moderate association with nocturnal sleep dysfunction in PD. Some pain subtypes had a greater effect on sleep than others.
Matei 2019	Iasi	Autonomic dysfunction,	To investigate autonomic cardiac control in PD	Case-control	30 patients with PD, 20 normal controls<br>Investigations: spectral analysis of short-	Low and high frequency were lower in PD patients than in controls (LF: 332. ±288.4 ms^2 PD vs 723.9±348.2 ms2 C; HF:

		peripheral nerve involvement	patients with normal serum levels of vitamin B12<br>To assess the prevalence of peripheral nerve disease		term heart rate variability (HRV).<br>Short-term ECG was used to calculate time domain and spectral parameters of HRV.<br>Electrophysiological examinations were performed in the motor fibers of median, peroneal and tibial nerves, and in the sensitive fibers of median and sural nerve.	283.72±241.97 ms² PD vs 530.54±226.5 ms² C, p<0.01). No differences between LF/HF ratio of PD and controls appeared.<br>Sensory nerve action potential in sural nerve was reduced in PD patients. No differences between sensory and motor nerve conduction velocities of PD and controls appeared.
Romosan 2019	Timisoara	Theory of mind	To measure affective theory of mind (ToM) abilities and cognitive performance in a sample of PD patients,	Case-control	65 PD patients and 51 healthy controls<br>Assessment: a visual affective ToM task (Reading the Mind in the Eyes – RMET), MoCA, and BPRS-E<br>(Brief Psychiatric Rating Scale).	Affective ToM abilities were preserved in early PD patients, declining as the disease progressed. Deficits in cognitive functioning predicted deficiencies in affective<br>ToM. Although attention, executive functions and visuospatial abilities together mediated the relationship between PD and affective ToM, only the impairment of visuospatial abilities had a specific negative impact on affective ToM. Moreover, 41% of the total effect of attention and executive functions on affective ToM was mediated by visuospatial skills.
Szasz 2019	Targu Mures	Clinical profile in advanced PD (APD)	To explor the profile of APD patients that were considered<br>and systematically evaluated regarding the suitability for device-aided therapies (DAT)	Retrospective	107 patients with APD that 1) described at least 2 hrs/day off periods divided into at least two instances /day (except early morning akinesia), 2) were in stage 3 or above on the H&Y scale, 3) were with or without dyskinesia, and 4) received at least four levodopa doses/day combined with adjuvant therapy.	Patients selected for DAT had significantly longer off<br>periods, more frequent dyskinesia, early morning akinesia, and freezing despite having significantly<br>higher LD doses than those with an improved conservative therapy.<br>PD patients should be considered for DAT when they receive a dose of LD of at least 750–1000 mg and maximal complementary therapies, and present daily motor complications that significantly reduce the quality of life.
Vanta 2019 a	Cluj-Napoca	Peripheral polyneuropathy	To investigate how or if associated polyneuropathy in PD affects health-related quality of life (HrQoL)	Observational, cross-sectional	73 non-demented PD patients<br>Investigations: Toronto Clinical Neuropathy Scale (TCSS), nerve conduction studies, and the Romanian version of PDQ-39.	36 patients presented a confirmed PN<br>Significant differences between mean scores in Motor, Activities of daily living and body discomfort domains of PDQ-39 in the PN-PD group versus non-PN group were observed.<br>TCSS significantly correlated to motor, emotional well-being and body discomfort domains.<br>The presence of associated PN in PD determines a further deterioration of HrQol in subjects with already<br>a poorer HrQol.
Vanta 2019 b	Cluj-Napoca	Peripheral polyneuropathy	To investigate the prevalence and features of the peripheral neuropathy in PD patients	Observational, cress-sectional	73 PD patients without a previously known cause of peripheral neuropathy (PNP)<br>Investigations: UPDRS II and III, Toronto Clinical Scoring System, biological evaluation (vitamin B12 and folic acid), and nerve conduction studies	The prevalence of PNP was 49.3%. In the L-Dopa group, the frequency of PNP was 67.3% as compared to PNP in the non-L-Dopa group.<br>The PNP was predominantly sensory with mild to moderate axonal loss. Cyanocobalamin correlated with<br>L-Dopa daily dose, and L-Dopa duration of administration.<br>L-Dopa daily dose correlated with the amplitudes of sensory nerve action potentials of the superficial

						peroneal and radial nerves.<br>The results imply that longer exposure to high doses of L-Dopa may cause vitamin B12 and folate imbalance and PNP, secondarily.
Attila 2020	Targu Mures	Gastrointestinal symptoms	To assess the gastrointestinal symptoms in the advanced stages of PD	Observational, retrospective	Authors analyzed the 6-year data of all levodopa-responsive patients with PD (n=286), with at least 2 hours per day off status, at least 3 on the Hoehn–Yahr scale during on phase, and at least four doses of levodopa per day.<br>Gastrointestinal symptoms were evaluated on the basis of yes/no answers given to questions about dysphagia, inappetence, epigastric discomfort, bloating, early satiety, nausea, vomiting and constipation.	The 181 patients with at least one gastrointestinal symptom had longer disease duration (10.13 ± 4.03 vs 7.4 ± 2.42 years), more severe clinical picture (longer off: 4.03 ± 1.32 vs 2.91 ± 1.02 hours, more lasting dyskinesia: 2.76 ± 0.91 vs 1.83 ± 0.61 hours, higher H&Y score) and received higher levodopa doses at higher dosing frequencies.<br>Constipation and bloating were the most common gastrointestinal complaints. Most of the complaints occurred more frequently in dyskinetic patients.
Ciopleias 2020 (poster)	Brasov	Autonomic dysfunction	To assess the prevalence of autonomic symptoms in male PD patients and their impact on quality of life.	Case-control	63 male PD patients and 63 controls.<br>The assessment of patients included: Non-Motor Symptoms Questionnaire and Scale, Parkinson's Disease Questionnaire (PDQ-39) and SCOPA-AUT scale	The PD patients had a mean age of 66.8±14.3 years, and a mean duration of PD of 6.2±4.7 years.<br>PD patients presented a higher prevalence for all autonomic domains, compared to control group ($p<0.05$). The prevalence was higher for drug-naïve patients and increased with age and disease severity.<br>The most affected domains were the urinary, gastrointestinal and sexual ones.<br>PD patients scored higher than controls in the total SCOPA-AUT score. Mean total SCOPA-AUT score was correlated with disease duration, disease severity and PDQ-39 scores
Diaconu 2020 (poster)	Brasov	Dietary habits, BMI	To evaluate dietary habits and food preferences of PD patients	Case-control	82 PD patients and 60 healthy controls<br>Assessment: standardized questionnaire that<br>included extensive food assessment and nutrient intakes, and dietary preferences.	Compared to healthy controls, PD patients had lower BMI and lower daily food intake.<br>PD patients reported a higher daily intake of the following nutrients: iron, potassium, carotene, and folate, but a lower daily intake of vitamin E.<br>PD patients consumed less alcohol and fewer calories. PD patients with dysphagia had softer food intake and used to drink less fluid per day. Regarding the diet style, the PD patients showed preferences for aliments belonging of Mediterranean diet. Protein intake was correlated with motor fluctuations.
Jozsef 2020	Targu-Mures	Gastrointestinal symptoms	Too assess the gastrointestinal symptoms in the advanced stages of PD	Retrospective, cohort	286 PD patients with at least 2 hours/ day off status, at least 3 on the H&Y scale during on phase, and at least 4 doses of levodopa / day.<br>Gastrointestinal symptoms were evaluated on the basis of yes/no answers given to questions about	The 181 patients with at least one gastrointestinal symptom had longer disease duration (10.13 +/- 4.03 vs 7.4 +/- 2.42 years), more severe clinical picture (longer off: 4.03 +/- 1.32 vs 2.91 +/- 1.02 hours), more lasting dyskinesia (2.76 +/- 0.91 vs 1.83 +/- 0.61 hours), higher<br>H&Y score) and received higher levodopa doses at higher dosing frequencies.

					dysphagia, inappetence, epigastric discomfort, bloating, early satiety, nausea, vomiting and constipation.	Constipation and bloating were the most common gastrointestinal complaints. Most of the complaints occurred more frequently in dyskinetic patients.
Muntean 2020 (poster)	Cluj-Napoca	Pain and sleep disturbances	To investigate the painful symptoms and night-time sleep problems in patients with PD and to determine the relation between pains and sleep disturbances	N/R	60 PD patients<br>Assessment: pain was measured on the visual<br>analogue scale (VAS) and using the Brief Pain Inventory (BPI). Nocturnal sleep was assessed using the Parkinson's Disease Sleep Scale (PDSS) and the quality of life was analyzed using the PDQ-39.	Pain was present in 63.33% of patients with a median severity of 6.5 on the VAS.<br>Night-time sleep was impaired in approximately 2/3 of the PD patients.<br>Authors found a correlation between higher scores on VAS and lower scores on PDSS, especially in the items assessing nocturnal motor symptoms (items 10-12).
Szasz 2020 (poster)	Targu-Mures	Gastrointestinal symptoms	To describe the spectrum of gastrointestinal complaints in patients with advanced Parkinson's Disease (APD), who were repeatedly evaluated to establish the opportunity of device aided therapy (DAT).	Retrospective	286 patients with APD<br>Assessment: questionnaires (yes-no responses).<br>The following gastrointestinal complaints were<br>queried: dysphagia, loss of appetite, epigastric discomfort, meteorism, early satiety, nausea, vomiting and constipation.	181 patients had at least one gastrointestinal symptom accompanied by significantly greater disease duration,<br>more severe clinical picture (longer off periods or more severe dyskinesia, higher H&Y score) and significantly higher doses of LD (both total daily dose and dosing frequency).<br>Symptoms suggesting gastroparesis (dysphagia, loss of appetite, epigastric discomfort, meteorism, early satiety, nausea, vomiting) were, without exception, significantly<br>more prevalent in patients with dyskinesia.

Table S2. Diagnostic accuracy studies.

Study	Region	Test	Objective	Type of study	Methods	Main results
Martinez-Martin 2009 a	Multicenter, Brasov	Non-Motor Symptoms Scale (NMSS)	To investigate the psychometric properties of NMSS	Cross-sectional, with retest	411 PD patients, from 12 centers across 10 countries<br>Assessment: NMSS, Scales for Outcomes in Parkinson's Disease (SCOPA)-Motor, SCOPA-Psychiatric Complications (SCOPA -PC), SCOPA-Cognition, H&Y staging, Clinical Impression of Severity Index for Parkinson's Disease (CISIPD), SCOPA -Autonomic, Parkinson's Disease Sleep Scale (PDSS), Parkinson's Disease Questionnaire– 39 items (PDQ-39), and EuroQol–5 dimensions (EQ-5D).	The NMSS score was 57.1 ± 44.0 points. The scale was free of floor or ceiling effects.<br>For domains, the Cronbach alpha coefficient ranged from 0.44 to 0.85.<br>The intraclass correlation coefficient (0.90 for the total score, 0.67– 0.91 for domains) and Lin concordance coefficient (0.88) suggested satisfactory reproducibility.<br>The NMSS total score correlated significantly with SCOPA-Autonomic, PDQ-39, and EQ-5D.<br>Association was close between NMSS domains and the corresponding SCOPA– Autonomic domains, and also with scales measuring related constructs (PDSS,<br>SCOPA-PC).<br>The NMSS total score was higher for women, and for<br>increasing disease duration, H&Y, and CISI-PD severity level.
Martinez-Martin 2009 b (poster)	Multicenter, Brasov	Non-Motor Symptoms Scale (NMSS)	To explore a PD staging based on non-motor symptoms (NMS) severity levels determined through NMSS.	Cross-sectional	750 PD patients<br>Assessment: NMSS, H&Y staging, motor examination and motor complications scales, and the PDQ-	NMSS total score was 56.82±43.62 (range: 0–243, median: 44).<br>NMSS levels were established as follows: level 0 (no NMS); 1 (slight): 1–7 points; 2 (mild): 8–24; 3 (moderate): 25–44; 4 (severe): 45–80; 5 (very severe): ≥81 points.

					8.<br>NMSS scores were broken down by quartiles to establish severity levels.	No differences were detected in NMS severity level distribution by gender and age. Disease duration, motor examination, motor complications, and PDQ-8 scores showed significant differences by NMSS severity levels. There was also a significant difference between H&Y and NMS levels.<br>Authors concluded that severity levels, based on quartiles, can be extracted from NMSS scores and may be the basis for a staging system based on NMS.
Martinez-Martin 2009 c (poster	Multicenter, Brasov	Scales for Outcomes in Parkinson's Disease-Cognition (SCOPA-COG)	To analyze the validity of the SCOPA-COG as a measure of cognitive deterioration in PD	Cross-sectional	308 PD patients<br>Assessment: H&Y staging, MMSE, Clinical Dementia Rating (CDR), Frontal Assessment Battery (FAB), Parkinson Psychosis Rating Scale (PPRS), Clinical Impression of Severity Index- Plus (CISI-PDp), and SCOPA-COG. Dementia was ascertained according to DSM-IV criteria. Acceptability, internal consistency, and construct validity were analyzed.	SCOPA-COG mean score was 18.1±10.2 (observed range: 0-43). No floor or ceiling effects were detected and the Cronbach's alpha coefficient for the complete scale was 0.92.<br>SCOPA-COG showed high correlations with MMSE, FAB, CDR, and CISI-PD Cognitive status.<br>SCOPA-COG score significantly decreased as PD (H&Y) and dementia (CDR) severity increased.<br>PD patients with dementia scored significantly worse in SCOPA-COG than those without.
Kraus 2011 (poster)	Multicenter	Spiralometry	To introduce an objective method for quantification of kinetic tremor	N/R	1703 early and advanced PD patients, treated with Pramipexole Kinetic tremor was evaluated by spiralometry - a fully standardized and fully automated technique for quantification of tremor from PD patients' hand-drawn spirals.	For all patient subgroups, the spiralometric measured tremor amplitude decreased significantly for both right and left hands from baseline to study end.<br>Spiralometry proved to be a sensitive technique to quantify tremor amplitude from hand-drawn spirals as well as a versatile and robust tool for application with large numbers of examinations and various ways of communication
Martinez-Martin 2011	Multicenter, Brasov	Movement Disorders Society Task Force (MDS-TF) criteria	To explore the utility of the new MDS-TF criteria and procedures in clinical practice.	Cross-sectional	299 PD patients (36.5% with PDD as per MDFS-TF criteria; 33.1% according the DSM-IV).<br>Assessment: a variety of standardized motor, cognitive, psychiatric, and global severity measures.	Agreement between MDS-TF and DSM-IV criteria was substantial (87.3%; kappa ¼ 0.72), but the DSM-IV criteria failed to identify 22% of patients fulfilling MDS-TF criteria.<br>False negative cases were older and had more severe motor symptoms but less psychosis than those true non-demented PD. False positives had less severe motor symptoms than true PDD, although the difference did not reach statistical significance.
Geman 2012	Suceava, Iasi	Time series analysis, including the wavelet transform	To identify new parameters / features of time series for a better pathological tremor classification.	N/R	28 PD (Parkinson's Disease tremor), 24<br>SPD ("Suspicious" PD tremor), and 30 NT (Normal tremor) subjects were analyzed.<br>All patients are suffering moderate to severe postural tremor.<br>The tremor time series were acquired using an accelerometer sen-	For PD and SPD tremor signals the high value coefficients were located between 200 and 1200 samples. The number of spikes within this region was more than 10. For the N tremor signal the number of spikes was smaller, but roughly with the same distribution over the analyzed samples.<br>Linear, nonlinear and statistical methods are not suitable for an early detection of PD. Considering different types of tremor signals, including from patients considered to be suspect of PD, it has been observed that there are strong similarities between the PD tremor

					sor from a Wii™ console, connected via Bluetooth™ to a<br>PC. The data were analyzed using an application implemented in Visual C 2010 Professional.	signals and suspect PD tremor signals after applying the wavelet transform.
Popa 2012 a	Iasi	Transcranial magnetic stimulation (TMS)	To investigate TMS in PD patients	Case-control	10 PD patients and 10 healthy controls<br>Investigation: single-puls TMS<br>Comparative analysis	Reduction of the electrical cortical silent period and a facilitation decrease in the motor areas corresponding to the upper limbs in PD patients
Popa 2012 b (poster)	Iasi	Transcranial magnetic stimulation (TMS)	To investigate by TMS the influence of functional electrical stimulation (FES) on cortical excitability	N/R	21 patients with PD<br>Investigation: single-pulse TMS at two different points in time: before and after 10 consecutive days of FES exercise for 30min/day applied to the radial nerve of the<br>most affected upper limb.	Changes in cortical excitability were noticed after<br>FES training. The motor threshold decreased by 6.5%, the motor evoked potential amplitude in hot spot increased by 33.2%, the contralateral silent period was longer by 31.2%, while latency dropped by 4.6% and duration by 4.9%.<br>A bilaterally broadening of the first dorsal interosseous muscle motor area, especially on the mediolateral axis, was observed.
Ungurean 2012	Suceava	Speech signals analysis	To use speech signal analysis in order to perform a preliminary prediction of PD	N/R	Authors used a dataset composed of a range of biomedical voice measurements from 31 people, 23 PD patients and<br>Optimization of Fuzzy clustering by Local Approximation of Membership (FLAME) algorithm on Cell BE processor	Authors propose the usage of FLAME clustering algorithm on the speech signals acquired from the PD patients. The algorithm has been optimized for CBEA-based processors in order to use intensive computing resources.<br>Classification accuracy was > 80%.
Alexa 2013	Iasi, Bucharest	Brainstem. auditory evoked potentials	To investigate the auditory brainstem pathways in patients with PD.	Case-control	34 patients with PD (H&Y = 2-4), 29 healthy controls.<br>Investigations: recordings of brain auditory evoked potentials (BAEP)<br>Averaged potentials to 1,000 clicks were obtained.	The BAEP results were interpreted for the latencies of waves I, II, III, IV, V and Interpeak Latencies (IPL) I-III, III-V and I-V. The results show that the waves II, III, IV, V and IPL III-V were significantly delayed bilaterally.<br>The changes did not correlate with the age or duration of disease.<br>The neurodegenerative process affecting the brainstem may determine modifications of auditory evoked potentials.
Geman 2013 a	Suceava	Intelligent optical sensors or accelerometers, specific nonlinear dynamics parameters or fuzzy logic	To investigate the use of different trainable Artificial Neural Networks for tremor analysis, which were based on different approaches to classify medical data sets by the<br>construction of Fuzzy Inference Systems or Fuzzy Expert Systems.	N/R	Authors used a database containing tremor measures from 58 patients with PD and 30 healthy subjects.<br>For the nonlinear analysis of tremor signals, authors used several software packages such as CDA (Chaos Data Analyzer Programs) and NLyzer (Nonlinear Analysis in Real Time).<br>CDA (Chaos Data Analyzer Programs) was used for nonlinear sig-	Authors present an accurate tremor and gait analysis system (Automatic Assessing Tremor Severity Systems) that is economical and noninvasive.

					nal analysis, and numerical parameters for tremor analysis.	
Geman 2013 b	Suceava, Iasi	Intelligent system for health status prediction using a Markov chain	To investigate if the Markov chains can be applied to the patients with PD in order to predict the evolution of the disease over time.	N/R	The authors used a dataset composed of a range of biomedical tremor measurements from 28 PD patients, 30 "normal" tremor and 30 "suspicious" PD (undiagnosed).<br>The intelligent system consists of 3 modules. The first module is used for the signal acquisition from patients suspected of PD (a software application). The second module is represented by the extracting knowledge from biomedical signals acquired from the patients (a software application). The third module is the application that is executed in the doctor's office.	Authors present a model of PD prognosis based on Markov process and show how this mathematical tool may be used to generate detailed and accurate assessments of PD stage and therefore may be applicable in medical screening for PD.<br>All clinically important events are modeled as transitions from one state to another.<br>The use of Markov models has the potential to allow the development of decision models that more faithfully represent clinical problems.
Geman 2013 c	Suceava	Fuzzy expert systems, nonlinear dynamics	To propose a new screening system for quantitative evaluation and analysis, designed for the early-stage detection of PD	N/R	Authors used a database with tremor recordings of 28 PD patients, 24 SPD ("Suspicious" PD tremor), and 30 NT ("Normal" tremor)<br>The tremor time series were recorded using an accelerometer sensor from a Wii™ console, connected via Bluetooth™ to a PC. The data were analyzed using an<br>application implemented in Visual C 2010 Professional.	Authors proposed the implementation of a Fuzzy Expert System for tremor analysis, in order to improve the diagnostic accuracy of PD.
Fazakas 2013	Targu Mures	Serum arylsulphatase A (ASA)	To investigate the possible interference of ASA activity with sulfatide deficiency causing the metabolic and neurodegenerative aspects of diseases such as PD	Case-control	14 PD patients, 15 patients diagnosed with<br>Diabetes Mellitus type 2, 44 patients undergoing hemodialysis and 12 patients<br>representing the healthy control group.<br>Investigations: ASA from peripheral blood	The mean values of the ASA substrate concentration differed significantly between the group of PD patients and healthy controls, denoting a lower enzymatic activity in PD patients.<br>Authors conclude that the ASA deficit may be a metabolic<br>factor of the clinical aspects of the disease.
Pohoata 2013	Suceava	High-Definition Television Video System (HDTV)	To propose the implementation of a high-definition video system in the early management of PD	N/R	Authors used a dataset composed of a range of tremor measurements for 82 individuals (28 PD patients, 24individuals with suspicious tremor, 30 individuals without	After testing HDTV system functionality, an alternative method was proposed for video sequences assessing in order to eliminate uncertainty and provide an adequate telediagnosis.

					tremor).<br>The HDTV was designed to record suspicious patients, on their inhabitancy,<br>in order to detect tremor and analyze its evolution.	
Geman 2014 a	Suceava, Iasi	Automatic assessment of tremor severity	To investigate the automatic assessment of PD tremor using nonlinear dynamics, artificial neural networks and neuro-fuzzy classifier	N/R	Authors used a database containing tremor measures from58 PD patients and 30 healthy subjects.<br>The study used Artificial Neural Networks (ANNs) and an Adaptive Neuro-Fuzzy Classifier with Linguistic Hedges (ANFC-LH) for medical datasets (tremor, handwritten text images) with the goal of automatic classification of subjects in "Parkinsonian" or "non-Parkinsonian" (healthy).	Authors propose a method using intelligent optical sensors or accelerometers for biomedical signal acquisition, and some specific nonlinear dynamics parameters or fuzzy logic in PD tremor analysis.
Geman 2014 b	Suceava	Model for recognition and monitoring of PD patients	To develop a space-time nonlinear adaptive system which fuses brain and gait information algorithmically<br>To propose a model for recognition and monitoring of PD	N/R	The dataset is composed of a range of tremor measurements from 82 PD subjects<br>and 62 normal subjects.<br>Task 1: Setting up an effective data acquisition protocol<br>Task 2: Estimating dynamic tremor using gait, EEG and other information<br>Task 3: Organizing a physiological knowledge -based system<br>Task 4: Fusion of gait, EEG, handwriting, video and other potential physiological information<br>Task 5: Introducing and implementing a suitable decision-making strategy for Parkinson's detection, recognition,<br>treatment and monitoring	Authors propose a model employing multi-modal data evaluated by a hybrid intelligent system that provide<br>sufficient support for treatment and evaluation of patient recovery/deterioration.<br>A web application was constructed in order to collect and<br>process data information from intelligent sensors, to support the medical specialist in diagnosis, to provide to patients an easier access to their health information and to facilitate interaction between doctor and patient
Chaudhuri, 2015	Multicenter	King's PD Pain Scale	To develop the first PD specific pain scale	Cross-sectional	178 PD patients with otherwise unexplained pain; 83 non-spousal non-PD controls<br>Assessments: H&Y classification, Scale for Outcomes in PD-Motor, Non-Motor Symptoms Scale, Clini-	Floor effect was observed in all domains. The difference<br>between mean and median King's PD Pain Scale total<br>score was less than 10% of the maximum observed<br>value. Skewness was marginally high (1.48 for patients).<br>Factor analysis showed four factors in the King's PD<br>Pain Scale, explaining 57% of the variance (Kaiser-

					cal Impression of Severity Index in PD, Hospital Anxiety Depression Rating Scale, EQ-5D-3L, PDQ-8, Parkinson's disease sleep scale-version 2 (PDSS-2), Wearing-Off Questionnaire 9, Visual analog scales (VAS) for pain severity and frequency.	Mayer-Olkin, 0.73; sphericity test).<br>Cronbach's alpha was 0.78, item-total correlation mean value 0.40, and item homogeneity 0.22.<br>Correlation coefficients of the King's PD Pain Scale domains and total score with other pain measures were high. Correlation with the Scale for Outcomes in PD-Motor, Non-Motor Symptoms Scale total score, and quality of life measures was high.
Onu 2015	Bucharest	Resting-state functional magnetic resonance imaging (rs-fMRI)	To investigate the intra- and internetworks connectivity in PD, in an effort to find rs-fMRI biomarkers for PD	Cross-sectional	27 PD patients and 16 healthy subjects.<br>Investigation: rs-fMRI data, Independent Component Analysis (ICA)	23 ICA maps were identified as components of neuronal origin. For intra-network connectivity changes, eight components showed a significant connectivity increase in patients; these were correlated with clinical scores and were largest for (sensori)motor networks. For inter-network connectivity changes, authors found higher connectivity between the sensorimotor network and the spatial attention network, and lower connectivity between anterior and posterior default mode networks (DMN), anterior DMN and visual recognition networks, as well as between visual attention and main dorsal attention networks, for patients as compared to healthy subjects.<br>The area under the Receiver Operating Characteristics (ROC) curve for the best predictor (partial correlation between sensorimotor and spatial attention networks) was 0.772.<br>These functional alterations were not associated with any gray or white matter structural changes.
Biundo 2016	Multicenter	MMSE, MoCA	To investigate which is best suited to assess cognition in clinical practice and most sensitive to cognitive decline	Retrospective cohort study	265 LBD patients, 197 PD patients without dementia (PDnD), 40PD patients with dementia (PDD), and 28 patients with dementia with Lewy bodies (DLB) from an international consortium<br>Investigations: MMSE and MoCA at baseline and 1-year follow-up (N = 153).	Percentage of relative standard deviation (RSD%) for the MoCA (21 %) was greater than for the MMSE (13 %) in the whole group. This difference was significant only in PDnD (11 vs. 5 %), but not in PDD (30 vs. 19 %) or DLB (15 vs. 14 %).<br>The 1-year estimation of change did not differ between the two tests in any of the groups (Cohen's effect \0.20 in each group).<br>MMSE and MoCA were equal in measuring the rate of cognitive changes over time in LBD. However, in PDnD, the MoCA was a better measure of cognitive status as it lacks both ceiling and floor effects.
Fiorenzato 2016	Multicenter	MMSE, MoCA	To determine if MoCA is more sensitive than the commonly used MMSE in detecting cognitive abnormalities in patients with probable progressive supranuclear palsy (PSP) and multiple system atro-	Cross-sectional	35 MSA, 30 PSP and 65 age, and education and gender matched-PD patients.<br>Assessment: MMSE and MoCA were performed on two separate occasions within 5–7 days and administered in the morning ON medication, in a random order.	The mean MMSE was higher than the mean MoCA score in each MSA, PSP, and PD. MoCA total score as well as its letter fluency subitem differentiated PSP from MSA and PD with high specificity and moderate sensitivity.<br>MMSE presented an overall ceiling effect for most subitems, except for the pentagon scores, where PSP did less well than MSA or PD patients.<br>PSP and MSA, similar to PD patients, may present normal MMSE and

			phy (MSA) compared with PD			reduced MoCA performance.<br>Overall, MoCA is more sensitive than MMSE in detecting cognitive impairment in atypical parkinsonism and together with verbal fluency would be a useful test to support PSP diagnosis.
Geman 2016	Suceava	Joint EEG - EMG signal processing	To classify the mental tasks from both EEG and EMG signals, using combined signal processing and machine learning	N/R	Combined signal processing and machine learning have been used for classification of the mental tasks from both EEG and EMG signals. The signals were acquired from 22 patients with PD, while they performed four different mental tasks.	Authors proposed a process for identification of the mental task classes with good specificity and accuracy
Toderean 2016	Iasi	Nonlinear Dynamic Tools	To evaluate the differences between healthy subjects (HS), PD subjects (PDS), and epileptic subjects (EBS - Epileptic subject between seizures and ES - epileptic subjects in seizure) by computing some linear, statistic and non-linear features, such as: correlation dimension, maximum Lyapunov exponent and Hurst coefficient	N/R	Authors applied rescaled range method to estimate the Hurst coefficient of the decomposed signals, with different types of wavelet transforms.	The results obtained using the maximum Lyapunov exponent did not highlight very well differences between the 4 groups analyzed (HS, PDS, EBS and ES) but using Hurst coefficient authors obtained a very good differentiation of EEG signals.<br>The results show that the Hurst coefficient is significant different for delta and theta rhythms extracted from the EEG signal in case of the healthy subjects compared with PDS or EBS subjects. The Hurst coefficient for HS has a value higher than 0.5 and for PD subjects or EBS has a value lower than 0.5. Hence, the non-linear parameters such as Hurst coefficient can help in EEG interpretation and in neurological disorders diagnosis.
Tohanean 2016	Cluj-Napoca	Transcranial sonographic examination	To evaluate the diagnostic utility of transcranial ultrasound in PD	Case-control	12 early PD patients (H&Y = 1-2); 10 control subjects<br>Investigation: transcranial ultrasound examination of the brainstem through the temporal window.<br>The anatomical structures were evaluated by bilateral measuring of the ultrasound parameters: diameter of the lateral ventricles (VL), the degree of echogenicity and size of SN, raphe appearance, the diameter of third ventricle.	The prevalence of changes in the dopaminergic structures in PD was high: bilateral hyperechogenicity of SN over limit values of 0.20 cm^2 (100% of patients with PD) and in no subject from the control group. The degree of SN hyperechogenicity was moderate in 60% of patients and severe in the remaining 40%.<br>From early PD stages, important sonographic changes can be identified, being a marker for premotor PD.
Badea 2017	Bucharest	Functional connectivity (FC) changes on fMRI	To study the associated functional connectivity (FC) changes on fMRI, in PD patients.<br>To systematically investigate the reproducibility of the PD-related FC changes		Three resting-state fMRI datasets of PD were compared:<br>(1) the NEUROCON rs-fMRI study of 27 PD patients and 16 normal controls (with 2 replicate scans per subject) of the Neurology Department of the University Emergency	ROI-pairs with significant group differences (NC versus PD) in functional connectivity were found in all three PD datasets. However, these changes seemed at first sight to be distinct in each dataset<br>While global PD-related functional connectivity changes were non-reproducible across datasets, authors identified a few individual brain region pairs with marginally consistent FC changes across all three datasets. However, training classifiers on each one of the three da-

			across independent validation datasets.		Hospital Bucharest (Romania),<br>(2) a dataset of 20 PD patients and 20 normal controls provided by the group of Tao Wu (China),<br>(3) a publicly available dataset of 91 PD patients and 18 controls of the Parkinson's Progression Markers Initiative (PPMI) study in the US.	tasets to discriminate PD scans from controls produced only low accuracies on the remaining two test datasets. Moreover, classifiers trained and tested on random splits of the same dataset (which are technically homogeneous) also had low test accuracies, directly substantiating disease heterogeneity.
Bajenaru 2018 (poster)	Bucharest	Vitamin D level and insulin resistance.	To investigate vitamin D level and insulin resistance in PD	Case-control	44 consecutive PD patients; 20 healthy controls<br>Investigations: the serum level of 1,25-dihydroxycholecalciferol, and that of insulin and glucose which have been used to calculate the HOMA-IR index	In both groups the average level of 1,25 dihydroxycholecalciferol was lower than the referential laboratory values, but there was no significant difference between the PD group and the control group.<br>There was no significant difference in the insulin resistance as measured by the HOMA-IR index between PD patients and controls.
Geman 2018	Suceava	Response Surface Methodology (RSM)	To develop a mathematical model that may be helpful for medical doctors and researchers for predicting and diagnosing the deep brain stimulation (DBS) results with a use of data mining techniques.	N/R	Authors acquired and processed a total of more than 100 records (trembling signals) from 16 PD subjects.<br>As the location of DBS brain stimulation, three targets were used: Ventro-intermediar nucleus (Vim), Globus Pallidus internal (GPi), Subthalamic nucleus (STN).<br>Both continuous and cyclic stimulation for both hemispheres, as well as bilateral stimulation, were used as a stimulus.	The statistical analysis showed that in the case of PD tremors the value of the Lyapunov exponent was the most relevant.<br>Authors conclude that non-linear dynamic parameter values can be used to differentiate the different types of tremor.<br>The results obtained with RSM guides provides a further step to find the best model for the kernel in which DBS will be made based on the input variables (tremor, frequency, and location amplitude).
Martinez-Martin 2018	Multicenter, Brasov	King's Parkinson's Disease Pain Questionnaire (KPPQ),<br>KPP Scale (KPPS).	To test the validity of KPPQ	Case-control	300 PD patients and 150 controls<br>First, a comparison between the KPPQ scores of patients and matched controls was performed. Next, convergent validity, reproducibility (test–retest) and diagnostic performance of the questionnaire were analyzed.	PD patients declared significantly more pain symptoms than controls.<br>The KPPQ convergent validity was high with KPPS total score, but weak or moderate with other pain assessments.<br>Test–retest reliability was satisfactory with kappa values ≥0.65 except for item 5, Dyskinetic pains, and the intraclass correlation coefficient (ICC) for the KPPQ total score was 0.98.<br>After the scores of the KPPS were adapted for screening (0, no symptom; ≥1, symptom present), a good agreement was found between the KPPQ and the KPPS (ICC = 0.88). There was a strong correlation between the two instruments.<br>The diagnostic parameters of the KPPQ were very satisfactory as a whole, with a global accuracy of 78.3%–98.3%.
Stocchi 2018	Multicenter	Parkinson's Disease	To validate the PDCS	Cross-sectional	194 PD patients<br>Assessments: PDCS, MDS-UPDRS,	PDCS mean and median values were close. Skewness values were into the criterion limits (-1 to +1). The complete range of scores was cov-

		Composite Scale (PDCS)			Parkinson's Disease Sleep Scale Version 2, Montreal Cognitive Assessment, the Scale for Evaluation of Neuropsychiatric Disorders in Parkinson's Disease and the Clinical Impression of Severity Index for PD (CISI-PD). For test-retest analysis, a second administration of the PDCS was carried out in 61 stable patients (as per the CISI-PD) in 7–14 days after the first evaluation.	ered for 14 of the 17 items (83.4%). A floor effect of 25.26% and 28.25% was observed in the complications and disability level dimensions due to the proportion of patients free of these difficulties. No relevant floor or ceiling effect was observed for the PDCS total score. The stability of the scale appeared excellent with most items meeting weighted kappa and intraclass correlation coefficient values >0.80. The convergent validity of the PDCS with corresponding scores of the MDS-UPDRS showed high correlation values. The internal validity was into acceptable limits, with the majority of values higher than the minimal 0.30 threshold. The standard error of measurement suggested a satisfactory precision.
Toderean 2018	Suceava	Hurst coefficients on EEG delta rhythm	To investigate the application of the Hurst coefficients on EEG delta rhythm in PD patients	N/R	22 PD patients Investigation: joint EEG and EMG signals acquired on several mental tasks	the Hurst coefficient applied to EEG signals, appeared to be a good marker for the early diagnosis of PD,
Balestrino 2019	Multicenter	Parkinson's Disease Composite Scale (PDCS)	To determine the presence of PD manifestations, their distribution according to motor subtypes, and the relationships with health-related quality of life (QoL) using the recently validated European Parkinson's Disease Association sponsored PDCS.	Cross-sectional	776 PD patients Frequency of symptoms was determined by the scores of items (present if >0). Using ROC analysis and Youden method, MDS-UPDRS motor subtypes were projected on the PDCS to achieve a comparable classification based on the PDCS scores. The same method was used to estimate severity levels from other measures in the study. The association between the PDCS and QoL (PDQ-39) was analyzed by correlation and multiple linear regression.	The frequency of PD manifestations with PDCS and MDS-UPDRS were overlapping, the average difference between scales being 5.5%. Using the MDS-UPDRS subtyping, 215 patients (27.7%) were assigned as Tremor Dominant (TD), 60 (7.7%) Indeterminate, and 501 (64.6%) Postural Instability and Gait Difficulty (PIGD). With this classification as criterion, the analogous PDCS-based ratio provided these cut-off values: TD subtype, ≥1.06; Indeterminate, <1.06 but >0.65; and PIGD, <0.65. The agreement between the two scales on this classification was substantial<br>PDCS total score cut-offs for PD severity were: 23/24 for mild/moderate and 41/42 for moderate/severe.<br>Moderate to high correlations between PDCS and PDQ-39 were obtained, and the four PDCS domains showed a significant independent influence on QoL.
Cuciureanu 2019	Iasi	MRI examination	To evaluate the efficacy of modern current drug therapy for PD by correlating clinical and imaging data.	Observational, longitudinal	99 PD patients were followed periodically, clinically and radiologically for a period of 3 years after diagnosis<br>The MRI investigation was repeated over 12 months.	MRI examination can be an important tool for differential diagnosis, etiology and the prognosis of these patients
Martinez-Martin 2019	Multicenter	Parkinson's Disease Composite Scale (PDCS)	To extensively evaluate the PDCS's clinimetric properties	Cross-sectional	776 PD patients<br>Assessment: PDCS, the UPDRS and the Clinical Impression of Severity Index for PD (CISI-PD).<br>Basic clinimetric attributes of the	The PDCS total score showed negligible floor and ceiling effects. Three factors (54.5% of the variance) were identified: factor 1 included motor impairment, fluctuations and disability; factor 2, non-motor symptoms; and factor 3, tremor and complications of therapy.<br>Cronbach's alpha was from 0.66 to 0.79. Inter-rater reliability showed

					PDCS were analyzed.	weighted kappa values from 0.79 to 0.98 for items and intraclass correlation coefficient values from 0.95 (Disability) to 0.99 (Motor and total score).<br>PDCS standard error of measurement and convergent validity with equivalent constructs of other measures were satisfactory (≥0.70).<br>PDCS scores significantly differed by H&Y stage.
Aldred 2020 (poster)	Multicenter	5-2-1 screening criteria	To investigate if fulfilling the Delphi expert consensus 5-2-1 criteria suggest advanced Parkinson's disease (APD).	Observational	Patients from the DUOdopa / Duopa in Patients with Advanced PD - a Global Observational Study Evaluating Long-Term Effectiveness (DUOGLOBE), a single-arm, post marketing, observational, long-term effectiveness study of levodopa-carbidopa intestinal gel (LCIG) for advanced PD.<br>A 6-month interim analysis, of data from n=139 APD patients<br>5-2-1 criteria: at least one of the following: ≥ five-times daily oral levodopa use, ≥ two daily hours with 'Off' symptoms or ≥ one daily hour with troublesome dyskinesia	Most (98%) enrolled patients fulfill ≥ 1 of the 5-2-1 criteria. These patients responded favorably to LCIG treatment. Safety was consistent with other LCIG studies.<br>In advanced PD patients, the 5-2-1 criteria generally aligns with clinician assessment.
Geroin 2020	Multicenter, Brasov	Degree of trunk bending	To analyze the association between degree of postural abnormalities and disability and to determine cut-off values of trunk bending associated with limitations in activities of daily living (ADLs), motor impairment, falls, and back pain	Retrospective	238 PD patients with ≥5° of forward trunk bending (FTB), lateral trunk bending (LTB) or forward neck bending (FNB).<br>The degrees were calculated using a wall goniometer (WG) and software-based measurements (SBM).	Authors found significant associations between modified H&Y stage, disease duration, sex, and limitation in ADLs, motor impairment, back pain intensity, and history of falls. Degree of trunk bending was associated only with motor impairment in LTB).<br>ROC curves showed that patients with LTB of 10.5° (SBM, AUC 0.626) may have moderate/severe motor impairment.<br>The severity of trunk misalignment did not fully explain limitation in ADLs, motor impairment, falls, and back pain

Table S3. Intervention studies.

Study	Region	Intervention	Objective	Type of study	Methods	Main results
Arseni 1973	Bucharest	Stereotactic surgery with ^{90}Y	To assess the effects of stereotactic surgery, with or without additional treatment with L-Dopa	Retrospective	496 PD patients operated on between 1966 and 1972 are analyzed from the clinical, etiopathogenic, operative and postoperative points of view.<br>120 patients were operated on both sides.	Good and very good results were obtained against tremor, good results against hypertonia, and medium or poor results with regard to akinesia. The addition of L-Dopa to the stereotaxic treatment raised the score of the good results to very good for hypertonia, and of the poor and medium results to good ones for akinesia.
Pendefunda	Iasi	L-dopa and aman-	To investigate the treat-	Observational	73 PD patients	L-dopa was administered to 47 patients in an average daily dose

1973		tadine	ment of PD patients with L-Dopa and amantadine		Assessment: clinical status, apparition of AEs	of 3 g, with very good results in 26 cases, and no effects in 9 cases. The drug especially influenced akinesia and rigidity, and to a lesser extent tremor. No significant differences were observed with respect to the clinical form or age of the disease. The effects of the treatment were favorable in mild and moderately severe cases. Side effects, especially gastrointestinal, were noted in 27 cases.<br>Amantadine was administered to 26 patients in an average daily dose of 300 mg. Very good and good results were obtained in 12 cases, satisfactory in 10 and no effect in 4 cases. The treatment especially influenced akinesia and rigidity. Signs of intolerance were observed in 5 cases.
Pendefunda 1975	Iasi	L-dopa and amantadine	To investigate the treatment of PD patients with L-Dopa and amantadine	Observational	73 PD patients	L-Dopa was administered to 47patients (average daily dose of 3 g); excellent and good results were obtained in 26 cases, satisfactory results in 12cases, and no result in 9 cases. The effects of the treatment were better in the cases of medium and mild forms. Side effects were observed in 27 patients, most frequently gastrointestinal symptoms.<br>Amantadine was administered to 27 patients (average daily dose of 300 mg). Excellent and good results were obtained in 12 cases, satisfactory results in 10 cases and no result in 4 cases. The drug improved cases with akinesia and rigidity. Signs of intolerance were noted in 5 patients.
Cinca 1973	Bucharest	Anticholinergics, amantadine, and L-dopa	To study the therapeutic action of anti-Parkinsonian drugs	Retrospective	400 PD patients<br>A standardized method of objective clinical examination was used to provide quantitative data on the improvements induced by the different treatments.<br>The primary analysis concerned the effects of the anticholinergics, amantadine, and L-dopa as single treatments. A secondary study was carried out on combinations of L-dopa and anticholinergics and of L-dopa and amantadine.	Results demonstrated the superiority of L-dopa monotherapy as compared with amantadine and the anticholinergics. They also show the potentiating effect produced by combining these drugs with L-dopa. Combined treatment gives better results than L-dopa alone at the 3 g dose level, and can in certain cases equal a 0.5-2.0 g increase in L-dopa above this 3 g level.
Minea 2001	Brasov	α-Dihydroergocryptine (DHEC)	To investigate whether chronic coadministration of DHEC alters the plasma pharmacokinetics of individualized treatments with L-dopa	Controlled, open-label, repeated-dose (L-dopa and DHEC), sequential two period	12 PD patients<br>Steady-state pharmacokinetics of plasma L-dopa under combined treatment were compared with those under treatment with L-dopa alone.	There was no evidence of increased exposure to L-dopa caused by concomitant treatment with DHEC. In contrast, additional treatment with DHEC reduced the overall exposure to L-dopa; the time of maximum plasma concentration and peak-to-trough fluctuation were not affected. The maximum plasma concentration was reduced to a similar extent, albeit not significantly.

				study with a single fixed sequence.		
Baltag 2003	Iasi	L-dopa	To investigate the side effects of L-dopa	N/R	Initially, authors enrolled 116 PD patients (1993-2002)<br>After 3 years: only 62 patients<br>After 5 years: 36 patients<br>After > 5 years: 30 patients	Most frequent secondary effects comprised chorea, dystonia, mental adverse reactions.<br>Each one of the patients followed-up >5 years developed one or more types of secondary reactions
Olanow 2004	Multicenter, Bucharest	Sarizotan	To assess the safety and efficacy of Sarizotan in PD patients with dyskinesia	Open-label, dose escalation study	51 PD patients<br>The study consisted of a 3-week titration phase, a 9-week maintenance phase, and a 2-week withdrawal phase.<br>Sarizotan was initiated at a dose of 2 mg bid in week 1, increased to 5 mg b.i.d. in week 2, and titrated to a maximal dose of 10 mg bid during week 3 depending on patient response and tolerability.	Sarizotan treatment induced significant reduction in dyskinesia and particularly in troublesome dyskinesia.<br>Several patients experienced worsening parkinsonism with the introduction of Sarizotan or with increasing doses. This adverse event led to the withdrawal of 6 patients, and necessitated a dosage reduction in 35 patients.
Ferreira 2007	Multicenter, Bucharest	Nebicapone	To investigate the effects of nebicapone, on L-dopa pharmacokinetics, COMT activity, and motor fluctuations in PD in comparison to placebo and entacapone.	Randomized, double-blind, placebo-controlled, 4-way crossover	19 PD patients treated with carbidopa / levodopa 3 to 7 times per day.; 4 treatment periods (6-9 days duration each)<br>Treatment: Nebicapone/entacapone/ placebo and carbidopa/levodopa doses were administered concomitantly. At the end of each period, a levodopa test was performed, and levodopa and 3-O-methyldopa levels and COMT activity were assayed.	After 75 mg nebicapone, 150 mg nebicapone, and 200 mg entacapone, levodopa area under the plasma concentration time curve significantly increased, and 3-O-methyldopa area under the plasma concentration time curve significantly decreased.<br>Peak COMT inhibition was similar between active treatments, but extent of COMT inhibition was more sustained with 75 and 150 mg nebicapone than with 200 mg entacapone.<br>After the levodopa test doses, ON time significantly increased 29 minutes with 75 mg nebicapone, 45 minutes with 150 mg nebicapone, and 16 minutes with 200 mg entacapone.<br>Patients' diaries showed a decrease in daily OFF time of 109 minutes with 75 mg nebicapone, 103 minutes with 150 mg nebicapone, and 71 minutes with 200 mg entacapone and an increase in daily ON time of 74, 101, and 74 minutes, respectively. Treatments were generally, well tolerated and safe; no relevant changes in liver function tests were reported.
Borgohain 2009 (poster)	Multicenter, Targu Mures, Timisoara	Safinamide	To evaluate the efficacy and safety of safinamide as adjunctive therapy to levodopa in patients with PD experiencing motor	Phase III, randomized, double-blind, placebo-controlled	Patients with mid/late-stage PD receiving a stable dose of L-dopa and other PD therapies but experiencing>1.5 hour/day OFF (6-month study).	594/669 patients completed the study. Addition of safinamide significantly increased ON time compared with placebo without increasing troublesome dyskinesia. Both doses induced significant reductions in daily OFF time, OFF time after first morning levodopa dose, and UPDRS III scores while ON.

			fluctuations.		After L-dopa stabilization (4 weeks), patients were randomized to safinamide (50 [n = 223] or 100 mg/day [n = 224]) or placebo (n = 222). Primary efficacy endpoint was increase in mean daily ON time (without troublesome dyskinesia).	The discontinuation rate, serious and non-serious adverse events (AEs) were similar across treatments. Common AEs (>5%) were cataract, back pain, dyskinesia, headache, worsening PD, and depression.
Szasz 2009 (poster	Multicenter, Targu-Mures	Safinamide	To evaluate the improvements in symptom severity and daily living with safinamide as adjunctive therapy to L-dopa in patients with mid/late-stage PD	Phase III, randomized, double-blind, placebo-controlled	594 patients, with PD of >3 years' duration, >1.5 hour/day OFF time, GRID-HAM-D score <17 (no mild or moderate depression).<br>After L-dopa stabilization (4 weeks), patients were randomized to safinamide (50 [n = 223] or 100 mg/day [n = 224]) or placebo (n = 222). Stable doses of other PD medications were permitted.	Compared with placebo, addition of safinamide 50 and 100 mg/day significantly improved CGI change and severity scores. Significant improvements versus placebo in UPDRS II, GRID-HAM-D score, PDQ-39 total score, and related subscales of emotional wellbeing, communication, and bodily discomfort, as well as a trend in ADL, were also seen with safinamide 100 mg/day. These improvements were associated with significant improvements in motor fluctuations without worsening dyskinesia in both safinamide dose groups.
Bhatt 2010 (poster)	Multicenter, Timisoara, Targu Mures	Safinamide	To evaluate the efficacy of safinamide (50 and 100 mg/day) as add-on therapy to stable L-dopa in PD patients with motor fluctuations	Phase III (post-hoc analysis)	Study 016: 669 PD patients<br>The post-hoc analysis evaluated changes (baseline to Week 24) in: UPDRS Part IV (complications of therapy) total scores and scores for items 32-35 (dyskinesia and dystonia) and 32-34 (dyskinesia) in the overall population and a subset of patients with >30-min troublesome dyskinesia at baseline; Dyskinesia Rating Scale (DRS) scores; and diary categories (On without dyskinesia, On with minor dyskinesia, On with troublesome dyskinesia, or Off).	Safinamide 50 and 100 mg/day significantly improved ON time without increasing troublesome dyskinesia vs placebo; there were no between-group differences in DRS or UPDRS IV scores for dyskinesia and/or dystonia.<br>Dyskinesia as an adverse event was reported more frequently in the safinamide groups; however, it was generally transient and mild/moderate in severity.<br>The patient diaries indicated no increase in ON time with troublesome dyskinesia.<br>In the post-hoc analysis of patients with >30-min troublesome dyskinesia at baseline who completed the study (n=186), safinamide 100 mg/day significantly improved UPDRS IV scores for items 32-34, items 32-35, and total score compared with placebo.<br>With the exception of ON time for safinamide 50 mg vs placebo, there were no between-group differences in diary parameters in the dyskinesia subset.
Kaufmann 2010 (poster)	Multicenter	Droxidopa	To evaluate the clinical benefit, safety and tolerability of droxidopa to treat symptomatic neurogenic orthostatic hypotension (NOH).	Phase III multinational, placebo-controlled, parallel group, induction-design study	142 patients with NOH (39% of patients with PD, 15% with multiple system atrophy, 33% with pure autonomic failure and 13% with other autonomic neuropathies)<br>Patients entered an open-label, forced titration to determine an optimal treatment dose of droxidopa ranging from 100 mg T.I.D. to	Data currently available from the open-label titration on the first 120 patients indicates a highly significant average improvement in standing systolic blood pressure of 23 mmHg measured during clinical testing.

					600 mg T.I.D in 100 mg T.I.D. increments.	
Meshram 2010 (poster)	Multicenter, Timisoara	Safinamide	To assess the efficacy of safinamide as add-on therapy to stable L-dopa	Study 016, a 24-week phase III trial,	594 patients with mid/late-stage PD receiving a stable dose of L-dopa and other PD therapies but experiencing>1.5 hour/day OFF (6-month study).<br>Assessment: diary ratings (ON, OFF, ON with troublesome/minor dyskinesia), UPDRS) Part II, III and IV, Dyskinesia Rating Scale (DRS) scores.<br>Post-hoc analyses assessed baseline to week 24 changes in UPDRS subscale scores.	Safinamide 50 and 100mg/day significantly improved ON time without increasing troublesome dyskinesia vs. placebo. UPDRS-III total and subscale scores<br>also showed that safinamide improved motor function.<br>Although dyskinesia as an adverse event (AE) was more frequent with safinamide, there were no significant between-group differences in UPDRS-IV (items 32-35) or DRS scores.
Vasile 2010	Bucharest	Cholinesterase inhibitors	To assess the comparative efficacy of cholinesterase inhibitors	Randomized	38 patients diagnosed with PDD, who presented a documented history of PD for at least 3 years, and randomized them on single-blinded flexible doses of galantamine (mean daily dose 16 mg, dose range 8-24 mg/day, n=11), donepezil (mean daily dose 7.5 mg, dose range 5-10 mg/day, n=10), rivastigmine (mean daily dose 9 mg, dose range 6-12mg/day, n=10) or gingko biloba (mean daily dose 120 mg daily, dose range 90-150 mg/day, n=7). Patients were evaluated every 4 weeks for 6 months using MMSE, Alzheimer Disease Assessment Scale- Cognitive subscale (ADAS-Cog), Global Assessment of Functioning (GAF) and UPDRS.	Patients with PDD had a better evolution under treatment with rivastigmine and galantamine, without significantly intergroup differences. Donepezil improved the clinical status, but less than the other two cholinesterase inhibitors, although the intergroup difference did not reach a level of significance of 0.05. The difference between cholinesterase inhibitors and gingko biloba efficacy was significant. The tolerability of galantamine and donepezil was superior to that of rivastigmine, but inferior to gingko biloba.
Borgohain 2011 (poster)	Multicenter, Targu-Mures, Timisoara	Safinamide	To assess long-term efficacy and safety of safinamide, as add-on to L-dopa.	Study 018, an 18-month, double-blind, placebo-controlled extension to Study 016	544 of the 669 patients from Study 016 entered Study 018<br>Primary endpoint was change from baseline (start of Study 016) to Month 24 in Dyskinesia Rating Scale (DRS) scores. Analysis was hierarchical; if primary endpoint was not met, secondary	At month 24, least squares mean changes compared with placebo were -0.51 (50mg/day) and -0.59 (100mg/day).<br>In patients with baseline DRS >4, safinamide 100mg/day improved DRS scores compared with placebo (post-hoc analysis).<br>Improvements were observed in various secondary endpoints, including ON time with no/minor dyskinesia.<br>Incidences of serious and clinically notable AEs were similar between groups.

					endpoints were exploratory.	
Meshram 2011 (poster)	Multicenter, Timisoara, Targu-Mures	Safinamide	To evaluate the long-term safety and tolerability of safinamide as add-on to L-dopa	Study 018, an 18-month, double-blind, placebo-controlled extension to Study 016	544 PD patients with motor fluctuations<br>Safety and tolerability were assessed using adverse events (AEs), laboratory, vital signs, ophthalmological and ECG data.	Discontinuations due to AEs, serious AEs and deaths over 2 years were similar between placebo and safinamide 50mg/day, and slightly higher with safinamide 100mg/day. The most common newly emergent AEs during Study 018 were PD, dyskinesia and cataract There were no clinically relevant differences in other assessments among groups.
Vasile 2012 (poster)	Bucharest	Cholinesterase inhibitors	To investigate if cholinesterase inhibitors are equally efficient in the treatment of PDD.	Randomized, single blinded	45 patients with PDD, who presented a documented history of PD for at least 3 years<br>Interventions: flexible doses of galantamine (mean daily dose 16 mg, dose range 8–24 mg/day, n=16), donepezil (mean daily dose 7.5 mg, dose range 5–10 mg/day, n=15) or rivastigmine (mean daily dose 9 mg, dose range 6–12 mg/day, n=14).<br>Assessment: every 4 weeks for 6 months, using MMSE, Alzheimer Disease Assessment Scale- Cognitive subscale (ADAS-Cog), Global Assessment of Functioning (GAF) and UPDRS.	Patients with PDD had a better evolution under treatment with rivastigmine and galantamine, without significantly intergroup differences.<br>Donepezil had a positive impact over the mental clinical status, but lesser than the other two cholinesterase inhibitors.<br>There was recorded no drop out due to adverse events during the study. The UPDRS scores didn't vary significantly, reported to baseline, in none of the three groups.
Popa 2013	Iasi, Cluj-Napoca	Functional electrical stimulation	To investigate the effects of functional electrical stimulation in PD patients as a supplement to L-dopa therapy	Cross-sectional	Investigations: clinical (using specific tests) and paraclinical (using single-pulse transcranial magnetic stimulation)	The study revealed an improvement in the motor symptoms and the bilateral activation of the primary motor areas of the upper limbs after unilateral functional electrical stimulation in PD patients.
Vasile 2013 (poster)	Bucharest	Bupropion	To evaluate the efficacy and tolerability of bupropion in the treatment of major depressive disorder associated to PD.		20 patients diagnosed with PD for at least 2 years, who were admitted to the hospital for a major depressive episode<br>Intervention: bupropion XL (flexible dose 150–300 mg daily) for 6 months.<br>Assessment: Montgomery Asberg Depression Rating Scale (MADRS), Clinical Global Impression- Improvement (CGI-I), Global Assessment of Functioning (GAF), Monitoring of Side Effects Scale	Patients treated with bupropion had a significant improvement in the depressive symptom's severity. There was an improvement of symptomatology.<br>The differences between the baseline and endpoint severity of PD symptoms were significant in the 'non-motor experiences of daily living' dimension of MDS-UPDRS. SF-36 showed an improvement of the scores not only on 'mental health' scale, but also on the 'general health', 'vitality' and 'social functioning'. The overall SF-36 score improved significantly when compared to baseline. The tolerability was good, with only mild (n = 4) and moderate (n = 3) side effects being reported. The most frequently reported side effects were anxiety, insomnia and sweating.

					(MOSES), UPDRS, and SF-36 questionnaire.	
Baetu 2014 (poster)	Bucharest	LCIG	To assess the benefit of LCIG in advanced PD	N/R	16 patients with levodopa/carbidopa intestinal gel pump were valuated from January 2012-december 2013.<br>Evaluation: disease duration, oral L-dopa dose, H&Y stage, gait, quality of life<br>At 6 months and 1 year: neuropsychological functions, UPDRS.<br>Authors described the adverse events and assessed daily levodopa doses before, 6 months and 1 year after treatment.	2 patients received 24-hour duodenal infusion of LCIG with benefic effect on nocturnal insomnia and motor fluctuations. None of them patients had severe complications.<br>There was a significant reduction in fluctuations and dyskinesias and important improvement of gait function and quality of life. A significant improvement of depressive symptoms was detected. Sleep quality and diurnal somnolence ameliorated. Mean Off time was significantly reduced at all time points. Similarly, On time without dyskinesia was improved at all time point.
Borgohain 2014 a	Multicenter, Targu Mures, Timisoara	Safinamide	To evaluate the efficacy and<br>safety of safinamide, as add-on to L-dopa in the treatment of patients with PD and motor fluctuations	Phase III, multicenter, double-blind, placebo-controlled, parallel-group	Patients were randomized to oral safinamide 100 mg/day (n=224), 50 mg/day (n=223), or placebo (n=222) for 24 weeks.<br>The primary endpoint was total on time with no or non-troublesome dyskinesia (assessed using the Hauser patient diaries). Secondary endpoints included off time, UPDRS Part III (motor) scores, and Clinical Global Impression-Change (CGI-C).	At week 24, there was an increase in total on time with no or non-troublesome dyskinesia for both safinamide groups.<br>The differences in both safinamide groups were significantly higher versus placebo.<br>Improvements in off time, UPDRS Part III, and CGI-C were significantly greater in both safinamide groups versus placebo.<br>There were no significant between-group differences for incidences of treatment emergent adverse events (TEAEs) or TEAEs leading to discontinuation.
Borgohain 2014 b	Multicenter, Targu Mures, Timisoara	Safinamide	To assess the long-term efficacy<br>and safety of safinamide as an add-on to L-dopa in patients with mid- to late PD and motor fluctuations	18-month multicenter, multinational, randomized, double-blind, placebo-controlled, parallel-group extension study to the pivotal phase III study, Study 016	After a 6-month study, further long-term safinamide use in these patients was evaluated over an additional 18 months. Patients continued on their randomized placebo, 50, or 100 mg/d safinamide. The primary endpoint was change in Dyskinesia Rating Scale total score during ON-time over 24 months. Other efficacy endpoints included change in ON-time without troublesome dyskinesia, changes in individual diary categories, depressive symptoms, and quality of life measures.<br>50 of the 594 patients who complet-	Change in Dyskinesia Rating Scale was not significantly different in safinamide versus placebo groups, despite decreased mean total Dyskinesia Rating Scale with safinamide compared with an almost unchanged score in placebo. Ad hoc subgroup analysis of moderate to severe dyskinetic patients at baseline (36% of patients) showed a decrease with safinamide 100 mg/d compared with placebo.<br>Improvements in motor function, activities of daily living, depressive symptoms, clinical status, and quality of life at 6 months remained significant at 24 months. Adverse events and discontinuation rates were similar with safinamide and placebo.

					ed<br>Study 016 did not enter Study 018. Overall, 65.8% of Study 016 patients completed the whole 2-year treatment period, and 80.9% of Study 018 patients completed the 18-month extension period.	
Hauser 2014	Multicenter, Constanta	Tozadenant (SYN115)	To assess the safety and efficacy of tozadenant as an adjunct to L-dopa in PD patients who have motor fluctuations on L-dopa	phase 2b, randomized, double-blind, placebo controlled, parallel-group, dose-finding clinical trial	337 PD patients with motor fluctuations (at least 2·5 h off -time per day) completed study treatment. Patients received tozadenant 60, 120, 180, or 240 mg or matching placebo twice daily for 12 weeks<br>The primary outcome was change from baseline to week 12 in hours per day spent in the off -state (assessed from Parkinson's disease diaries completed by patients).	Compared with placebo, mean daily off -time was significantly reduced in the combined tozadenant 120 mg twice-daily and 180 mg twice daily group. The most common adverse events in these groups were dyskinesia, nausea and dizziness. Tozadenant 60 mg twice daily was not associated with a significant reduction in off -time, and tozadenant 240 mg twice daily was associated with an increased rate of<br>discontinuation because of adverse events.
Baetu 2015 (poster)	Bucharest	LCIG	To report on the complications observed in the patients with advanced PD treated with LCIG	N/R	28 PD patients	Technical problems with the infusion device were present in 30% of patients. The most frequent problems were related to intestinal tube placement<br>LCIG had symptom relieving and stabilizing effects, without severe side effects.
Trenkwalder 2015	Multicenter	Prolonged-release oxycodone–naloxone (OXN PR)	To investigate the analgesic efficacy of OXN PR in patients with PD and chronic, severe pain.	Phase 2, randomized, placebo controlled, double-blind	202 PD patients (H&Y stage II–IV), with at least one type of severe pain, and an average 24-h pain score of at least 6 (assessed on an 11-point rating scale from 0=no pain to 10=pain as bad as you can imagine).<br>Intervention: oral OXN PR (n=92) or placebo (n=109) for 16 weeks (starting dose oxycodone 5 mg, naloxone 2·5 mg, twice daily).<br>The primary endpoint was average 24-h pain score at 16 weeks in the full analysis population.	The full analysis population consisted of 88 patients versus 106 patients.<br>Least squares mean average 24-h pain score at 16 weeks in the full analysis population was 5.0 in the OXN PR group versus 5.6 in the placebo group. Similar proportions of patients in each group had adverse events (65% vs 70%), treatment-related adverse events (57% vs 57%), and serious adverse<br>events (5% vs 6%). Treatment-related nausea was more common in the OXN PR group than in the<br>placebo group (17% vs 9%), as was treatment-related constipation (7% vs 6%).
Bajenaru 2016	Bucharest, Targu-Mures, Oradea, Cluj-Napoca,	LCIG	To report changes in motor complications after initiation<br>of LCIG therapy.<br>To determine the impact of LCIG therapy on the	Retrospective	A retrospective analysis (2009–2013) of LCIG therapy and the experience in nine neurology centers in Romania was performed (113 patients).<br>The impact of LCIG therapy was	There was significant improvement in PD symptoms after initiation of LCIG therapy. The ''on'' period increased, with a mean value of 6.14 h, and the dyskinesia period was reduced, with a mean value of 29.4 %. The quantified non-motor symptoms subsided. The patients exhibited significant improvements in QoL scores. There were few AEs and few cases of LCIG

	Iasi, Timisoara		daily L-dopa dose variation before/and after LCIG, to collect patient self-assessments of quality of life (QoL), and to study the overall tolerability and safety of LCIG administration.		evaluated by analyzing changes in motor fluctuations, dyskinesia and the patients' QoL after initiating therapy. The safety of LCIG therapy was estimated by noting agent-related adverse events (AEs) and medical device-related AEs.	therapy discontinuation.
Craciun 2016	Cluj-Napoca	L-Dopa	To evaluate the activity of superoxide dismutase (SOD) and glutathione peroxidase (GPx), and to investigate if these activities are correlated with the daily L-DOPA dose.	Case-control	18 PD patients receiving a mean L-DOPA dose of 352.94±175.41 mg/day and 16 healthy control subjects<br>Investigations: venous blood was collected after overnight fasting, for the assay of SOD activity, and the assay of GPx activity.	SOD levels were significantly decreased in patients group compared to controls. GPx activity was similar in subjects with PD patients compared to controls.<br>There was no significant correlation between the L-DOPA dose and the SOD or GPx activities.<br>A positive and statistically significant correlation was observed between the H&Y staging and the daily L-DOPA dose.
Dogaru 2016	Cluj-Napoca	Rehabilitation	To assess the clinical efficiency of natural therapeutic factors in Baile Tusnad in order to continue the rehabilitation treatment of patients with PD in a spa and climatic resort.	Prospective, longitudinal	17 PD patients (H&Y stages 1-3)<br>Intervention: rehabilitation treatment consisting of carbonated mineral water baths for 15 minutes, aerotherapy for 30 minutes daily, massotherapy, kinesiotherapy, performed daily for 16 days. Assessment: the Tinetti Gait and Balance Scale, the 10-m walk test, the Webster Scale, the Quality-of-Life Scale, adverse reactions.	At the end of treatment, there was an improvement of the walking distance and speed. There were statistically significant improvements of gait and balance, the Webster Scale scores, and the Quality-of-Life Scale scores. There were no side effects to the treatment applied.
Kenney 2016 (poster)	Multicenter, Constanta	Tozadenant	To analyze all dyskinesia data from a Phase 2b trial of fluctuating PD patients in order to understand the overall impact a study drug has on dyskinesias as measured by multiple endpoints, the temporal pattern of change, and whether the endpoints used to measure dyskinesias are consistent with one another.	N/R	All analyses were derived from a Phase 2b study of tozadenant in fluctuating Parkinson patients (Hauser et al. 2014). Dyskinesia measures were collected at baseline, Week 2, 6, and 12 for placebo and four active treatment arms including adverse events, patient reported diaries, and UPDRS Items 32, 33, and 34.	Overall AE reporting for incidence of dyskinesias was 8.3% for placebo, compared with 14.1%, 15.9%, 20.0%, and 20.2% for tozadenant 60, 120, 180, and 240 mg BID, respectively. Analyzing by time the imbalance between placebo and tozadenant 60mg and 120mg BID was early and transient while the imbalance between placebo and the 180mg and 240mg BID treatment arms persisted. Patient reported diaries demonstrated a dose response such that ON time with troublesome dyskinesia was not increased in the lower dose arms (60mg & 120mg BID) but was increased in the higher dose arms (180mg & 240mg BID) compared to placebo.<br>UPDRS Items 32, 33, and 34 indicated no difference in dyskinesias between placebo and active treatment arms. The measures used to quantitate dyskinesias were inconsistent with one another.
Rizos 2016	Multicenter,	Short- and long-	To assess the occurrence of	Survey based	425 PD patients	ICD frequencies (as assessed by clinical interview) were signifi-

	Brasov	acting dopamine agonists (DA)	impulse control disorders (ICDs) in PD patients treated with short- or long-acting DAs	on medical records and clinical interviews	Survey based on medical records and clinical interviews of patients initiating or initiated on DA treatment (both short- and long-acting, and transdermal): ropinirole (ROP); pramipexole (PPX) and rotigotine skin patch (RTG)	cantly lower with RTG compared with any other assessed DAs except for prolonged release PPX (PPX-PR). The rate of ICDs for PPX-PR (6.6%) was significantly lower than for immediate release PPX (PPX-IR). Discontinuation rates of DA therapy due to ICDs were low.
Antonini 2017	Multicenter	Levodopa-carbidopa intestinal gel (LCIG)	To evaluate the 24-month safety and efficacy of LCIG treatment in advanced PD patients under routine clinical care	Prospective for treatment-naïve patients (60% of patients) and partially retrospective for patients with ≤1 2 months of pre-treatment with LCIG (40% of patients).	375 PD patients were enrolled, and 258<br>patients completed the registry.<br>Assessment: motor fluctuations, dyskinesia, non-motor symptoms, quality of life, and safety	LCIG treatment led to significant reductions from baseline in “Off” time (hours/day), “On” time with dyskinesia (hours/day), Non-Motor Symptom Scale total, and individual domains scores, and Parkinson's Disease Questionnaire-8 item total score.<br>Adverse events deemed to have a possible/probable causal relationship to treatment drug/device were reported in 194 (54%) patients; the most frequently reported were decreased weight (6.7%), device related infections (5.9%), device dislocations (4.8%), device issues (4.8%), and polyneuropathy (4.5%).
Attila 2017	Tirgu-Mures	Selective monoamine oxidase B inhibitors	The evaluation of therapeutical strategies used in the neurology clinics of Tirgu Mures County Emergency Clinical Hospital in order to define the role of monoamine oxidase B inhibitors	Retrospective	From the 2194 reports authors used data focusing on the therapeutic recommendations.<br>Regarding disease duration, the patients were divided in two groups: less ≤ 5 years and > 5 years.	From the 1183 patients in first group, 243 received monoamine oxidase inhibitors (12 as monotherapy, 52 together with dopamine agonists, in 61 cases combined with levodopa. In 118 cases monoamine oxidase inhibitors were combined with levodopa and dopamine agonists. From 582 cases with PD for more than 5 years, 195 received monoamine oxidase B inhibitors (selegiline: 10 cases, rasagiline: 185 cases). In 429 cases authors did not find accurate data regarding disease duration (selegiline: 5 cases, rasagiline: 93 cases).<br>The use of monoamine oxidase B inhibitors was similar to those found in literature
Chaudhuri 2017 (poster)	Multicenter	LCIG	To present the design of and baseline patient characteristics from an ongoing global study assessing the long-term effectiveness of LCIG treatment in advanced PD in routine clinical care.	Single-arm, open-label observational study	Approximately 200 patients from over 50 centers will be enrolled.<br>The present analysis – 29 PD patients.<br>Inclusion: advanced PD patients treated with LCIG over 3 years in a routine clinical care setting (DUO-GLOBE)<br>The primary efficacy outcome will be the mean change from baseline to 36 months inpatient-reported	The current study is designed to provide a better understanding of the long-term effectiveness profile of LCIG for the treatment of advanced PD.

					"Off" time. Secondary endpoints will include dyskinesia duration (MDS-UPDRS items) and severity (MDS-UPDRS IV items 33+34 and the Unified Dyskinesia Rating Scale), activities of daily living (MDS-UPDRS II), motor function (MDS-UPDRS III) and fluctuations (MDS-UPDRS item 39), QoL (8-item Parkinson's Disease Questionnaire), and NMSS, specifically including sleep/daytime sleepiness assessed with the NMS Scale, Parkinson's Disease Sleep Scale (PDSS-2) and the Epworth Sleepiness Scale. Caregiver burden, measured with the Modified Caregiver Strain Index, and healthcare resource utilization will also be examined.	
Muller 2017 (poster)	Multicenter, Ramnicu Valcea	Rotigotine transdermal patch	To describe real-world management of PD with dopaminergic treatments and the influence of routinely used management strategies on clinical outcomes.	Prospective, non-interventional post-authorization safety study	1531 PD patients requiring either monotherapy (rotigotine /other DAs /L-dopa), or L-dopa in combination with rotigotine/ other DA, were included and followed for ≤33 months.<br>Primary safety objective: evaluation of cardiovalvular fibrosis.<br>Primary efficacy variable: change from baseline in UPDRS-III (motor), assessed by treatment received at a particular post-baseline visit.	Discontinuation reasons: lost to follow-up (10.1%), consent withdrawn (6.2%), adverse events ([AEs] 3.6%), other (8.2%).<br>0.2% patients experienced AEs of structural cardio-valvular pathology.<br>Mean UPDRS-III score numerically improved when assessed by treatment received at Month 15, and in most treatments received at Month 33
Szasz 2017	Targu-Mures	Selective monoamine oxidase B inhibitors	To evaluate the therapeutical strategies used in the neurology clinics of Tirgu Mures County Emergency Clinical Hospital in order to define the role of monoamine oxidase B inhibitors.	Retrospective	2194 records (2003-2016)<br>Two groups: ≤5 years disease duration (n=1183) and > 5 years (n=582)	In the group with ≤ 5 years of diasease duration, 243 received monoamine oxidase inhibitors: 12 as monotherapy, 52 together with dopamine agonists, in 61 cases combined with levodopa. In 118 cases monoamine oxidase inhibitors were combined with L-dopa and dopamine agonists.<br>In the group with disease duration of > 5 years, 195 received monoamine oxidase B inhibitors (selegiline: 10 cases, rasagiline: 185 cases).
Muller 2018	Multicenter, Ramnicu Valcea	Rotigotine transdermal patch	To obtain information on real-world management of PD with dopamine-	Prospective, non-interventional, multiple-	1531 PD patients under monotherapy with a dopamine agonis (DA), or L-dopa in combination with a DA were followed for ≤ 33 months;	Mean motor scores improved for all dopamine-substituting treatments. Patients with more severe motor-symptoms/increased disability were more likely to receive L-dopa alone or in combination with a DA at study

			substituting drugs.	cohort, post-authorization safety study		onset. More patients who started on combination therapy with L-dopa remained on this treatment versus those starting on DA monotherapy. Cardio-valvular pathology was rare and not found to be causally-related to rotigotine.
Attila 2019	Tirgu-Mures	Dopamine agonists	To evaluate the use of dopamine agonists as a therapeutic option among PD patients admitted to the Neurological Clinics of Tirgu Mures during15 years	Retrospective, cross-sectional	Authors investigated the data of all PD patients (2379 cases) treated in their clinics between 2003 and 2017. Analysis of the particularities of dopamine agonists' usage based on the therapeutic recommendations from the final report of these patients.<br>Regarding time since the diagnosis, the patients were divided in two groups: less ≤ 5 years and > 5 years.	From the 1237 patients with disease duration ≤ 5years, 665 received dopamine agonists (120 as monotherapy, 83 together with monoamine oxidase inhibitors and in 234 cases associated with L-dopa). The remaining 228 patients were treated with a triple combination of L-dopa, dopamine agonists and monoamine oxidase inhibitors. In patients suffering from PD for > 5years, in 364 cases out of 653, a dopamine agonist was part of the therapy.<br>The usage of dopamine agonists was similar to the data presented in other studies.
Breda 2019	Cluj-Napoca	The Mozart effect	To compare the results of the Mozart effect associated with specific kinetic treatment in the rehabilitation of PD and the effect of standard kinesiotherapy	Prospective case-control study	16 patients with PD, H&Y stages I-III, included in a motor neurorehabilitation program. The patients were assigned to two groups, a control group (n=8) with specific physiotherapy and the other group receiving the same specific kinetic treatment associated with the Mozart effect (n=8), for 2 hours daily, over 14 consecutive days. The patients were assessed using the Schwab & England scale (for the degree of dependence) and the PHQ-9 scale (for depression) before and after therapy.	After 2 weeks of therapy, the patients in the study group had much more obvious, statistically significant improvements in both scores (Schwab & England scale and the PHQ-9 scale).
Criciotoiu 2019	Craiova, Bucharest	L-dopa	To evaluate the possible correlation between routes of administration of L-dopa in patients diagnosed with PD, and the presence of digestive symptoms	Case-control	31 patients diagnosed with advanced PD (group A, with 14 patients that were treated with oral L-dopa and group B, with 17 patients treated with LCIG)<br>Assessment: UPDRS part 3 (motor part), 8-item Parkinson’s Disease Questionnaire (PDQ-8), Non-motor Symptoms Questionnaire for PD (NMS Quest)	The frequency of digestive symptoms was higher in patients with oral medication, especially vomiting / nausea, constipation and the unsatisfactory voiding of bowel.
Fasano 2019 (poster)	Multicenter, Targu Mures	LCIG	To investigate the utilization of monotherapy and combination therapies in	Real-world data	COSMOS study<br>409 APD patients treated for at least 12	Mean LCIG treatment duration was 35.7 (range:12.0–139.3) months with 9.7% of patients (n = 39) on 24 h/day LCIG infusion.

			advanced PD (APD)patients during LCIG treatment		months (M) by the same physician since LCIG initiation.<br>Patients were grouped into LCIG monotherapy (LCIG the only treatment for PD), LCIG monotherapy during infusion hours (allowing add-on PD medications after LCIG infusion was stopped), and those on LCIG +add-on therapy.	Preliminary analyses indicate that the percentage of patients on LCIG monotherapy and LCIG monotherapy during infusion hours increased from LCIG initiation to M12, while the percentage of patients on LCIG + add-on therapy decreased at M12. A majority of patients discontinued the use of add-on PD medication at LCIG treatment initiation.
Nemes 2019	Cluj-Napoca	Dance therapy	To evaluate the effect of dance therapy on the quality of life, functionality and depression in PD patients.	Case-control	10 PD patients<br>Intervention: 40 minutes/day of dance therapy during two weeks.<br>Controls: 5 PD patients which did not attended dance classes.<br>All patients followed standard therapy: kineto-therapy and ergo-therapy.<br>Assessment: Beck Depression scale, Parkinson Disease Questionnaire (PDQ-39) and a satisfaction questionnaire developed by authors for the dance therapy.	In the group of dance therapy, there was an improvement of depression symptoms and an increase in the quality of life.<br>The mean of satisfaction questionnaire for dance therapy in the study group was 39,70 from a maximum of 48 points.
Petrescu 2019 (poster)	Bucharest	Quetiapine	To compare the efficacy and safety of quetiapine in parkinsonian dementia (PDD) patients with and Alzheimer disease (AD) patients	Parallel group trial	36 patients, diagnosed with AD (n=18) and PDD (n=14) and psychosis symptoms were assigned to receive quetiapine for 8 weeks (with assessments every 2 weeks).<br>Assessment: MMSE, Brief Psychiatric Rating Scale (BPRS), the Clinical Global Impression Scale–Severity Subscale (CGI-S), UPDRS III, and the Abnormal Involuntary Movement Scale (AIMS).	The mean BPRS score decreased until the week 4 in both groups with no statistical difference.<br>After 4 weeks, improvements slowed and continued without a significant decrease. The mean CGI-S score significantly decreased until the eighth week.<br>The median MMSE score was higher at the end point than at the baseline. Motor conditions remained unchanged after taking quetiapine in both groups. Dyskinesias decreased significantly only in AD group. Side effects were mild, generally transient, and well tolerated.
Szasz 2019 a	Targu-Mures	Therapeutical strategies	To analyze the therapeutical choices during the early phase of PD	Retrospective, cross-sectional	During 15 years, 2379 patients with PD were hospitalized, and 1237 patients had a disease duration < 5 years.	In the group with disease duration < 5 years, 18 patients had monoamine oxidase inhibitor monotherapy. Also, 665 patients received dopamine agonists (120 cases as monotherapy and in 83 patients associated with monoamine oxidase inhibitors)<br>521 patients received only L-dopa treatment. A further 481 patients received combined therapy (L-dopa with dopamine agonists and/or monoamine oxidase inhibitors).
Szasz 2019 b	Targu-	L-dopa	To analyze substitution	Retrospective	Data from all patients with ad-	125 patients were proposed device-aided therapies whereas in 42

	Mures		therapy in patients with advanced PD		vanced PD hospitalized between 2011-2017 (n=311), receiving combined L-dopa treatment at least 4x/day, reporting a minimum of 2 hours off periods, with or without dyskinesia.	patients the L-dopa dose was increased. The average L-dopa doses and the administration rate were higher for the 107patients tested for LCIG. Disease duration, mean L-dopa doses and frequency of dosing were all higher in patients proposed for device-aided therapies versus patients with continued conservative treatment.
Szasz 2019 c	Targu Mures	Dopamine agonists (DAs)	To evaluate the use of DAs as a therapeutic option among PD patients	Retrospective	Data from the records of PD patients (2003-2017).<br>Authors analyzed the particularities of DA usage based on the therapeutic recommendations from the final report of these patients.<br>Regarding time since the diagnosis, the patients were divided in two groups: ≤ 5 years(n=653) and > 5 years (n=1237).	In the group with a disease duration ≤ 5 years, 665 received DAs: 120 as monotherapy, 83 together with monoamine oxidase inhibitors and in 234 cases associated with L-dopa. The remaining 228 patients were treated with a triple combination of L-dopa, DAs and monoamine oxidase inhibitors.<br>In the group with a disease duration > 5 years, in 364 cases, a DA was part of the therapy.
Constantin 2020	Targu-Mures, Timisoara	LCIG	To analyze the causes that led to the discontinuation of LCIG therapy.	Retrospective, observational	After 10 years of experience with LCIG as<br>a therapeutic option in advanced PD, the authors analyzed the data of all dropout cases among the 204 patients that initiated LCIG therapy	43 patients dropped out.<br>In dropout patients, the disease duration until LCIG infusion was significantly longer and the overall clinical picture more sever (both regarding motor symptoms and cognitive decline), compared to patients who continued treatment. The dropout patients also presented significant differences regarding the incidence of polyneuropathy. The main cause of discontinuation was death.
Fasano 2020 (poster)	Multicenter, Targu-Mures	LCIG	To analyze the motor symptoms in patients with APD on long-term LCIG monotherapy<br>or combination therapy	Retrospective, cross-sectional, post-marketing, observational	378 APD patients treated with LCIG for ≥12 months. Patients were stratified into 3 groups: LCIG monotherapy (n = 120), LCIG daytime monotherapy with oral or transdermal PD medication at nighttime only (n = 94), and LCIG plus add-on PD medications (n = 164).<br>Assessment: motor symptom frequency / severity, and evaluation of “Off” time and “On” time with dyskinesia before starting LCIG and at study visit.	Patient characteristics were similar between groups. Patients treated with LCIG monotherapy tended to have slightly lower baseline “Off” time and dyskinesia duration than other treatment groups.<br>All treatment groups experienced significant reductions from baseline in “Off” time and dyskinesia duration, with no significant between-group differences. Most motor symptoms showed improvements in frequency and severity after LCIG initiation.
Popa 2020 a	Cluj-Napoca	LCIG and oral L-dopa	To study the effects of Levodopa -Carbidopa therapy in 2 separate groups: one with LCIG and the second with oral therapy.	Retrospective, observational	61 PD patients (H&Y stage 3 and 4)<br>Intervention: LCIG (n=24), oral L-dopa (n=37)	The mean adjusted UPDRS III (and similarly for UPDRS II) improved in the LCIG compared to the oral therapy group. There was a 41.7% (10) reduction in dyskinesia, and 29.2% reduction in wearing off/on-off at 1 year in the LCIG group compared to 0% (0)<br>dyskinesia reduction, and 2.7% reduction in wearing off/on-off in the oral therapy group.

						Continuous intrajejunal infusion of LCIG ensures a significant and clinical reduction in motor fluctuations compared to oral therapy in advanced PD, even after adjustment for important confounders.
Popa 2020 b	Cluj-Napoca	LCIG	To investigate the percutaneous endoscopic transgastric jejunostomy (PEG-J) use and the occurrence of procedural complications in a group of PD patients, receiving LCIG therapy.	Retrospective, observational	24 PD patients (H&Y stage 3 and 4), under LCIG therapy.	PEG-J replacement was found in 54.2% of the patients. Erythema and inflammation at the level of stoma (37.5%) and allergic reactions (20.8%) were the most common complications. The most frequent reason for PEG-J replacement was tube dislocation (20.8%), and rarely the plied tube, the excess of adipose tissue, or bezoar (4.3% each). A third of the patients received PEG-J therapy during the night, after a median time of three years, after PEG-J initiation. A quarter of all the subjects had their LCIG therapy interrupted (median time until interruption - 1 year). The main discontinuation reasons were patient, or patient's relative refuses.
Rizos 2020	Multicenter, Brasov	Non-ergot oral and transdermal dopamine agonists (DAs)	To investigate the tolerability in PD patients treated with long-acting and transdermal DA (Rotigotine skin patch, Ropinirole extended release, or Pramipexole prolonged release)	Medical record-based, retrospective data capture, survey	425 PD cases<br>Assessment: medical records, and clinical interview-based follow-up survey of patients initiating or initiated on DA treatment (short and long acting) in a real-life setting.	Tolerability was above 90% irrespective of age, with no significant differences between younger and older patients.<br>Authors suggest that long-acting/transdermal DA are tolerated in non-demented older patients, as well as in younger patients, however, with lower daily dose in older patients.
Szasz 2020	Targu-Mures, Cluj-Napoca, Timisoara, Bucharest	LCIG	To document 24-month efficacy, safety and the effect on patient's quality of life (QoL) of long-term treatment with LCIG in advanced PD in routine clinical care.	Observational	GLORIA registry (2010-2015): 39 advanced PD patients<br>Follow-up to 24 months	Results for Romania (GLORIA registry):<br>During the study period LCIG led to significant improvements in "Off" time, "On" time with dyskinesia, activities of daily living (ADL), motor examination, non-motor symptoms, and QoL which maintained up to the end of follow-up. At 24 months, "Off" time had a mean reduction of -5.2±3.1 hours/day vs. baseline and "On" time with dyskinesia had a mean reduction of -3.5±3.3 hours/day vs. baseline. Both ADLs and motor examination "On" scores showed a maximum improvement at 6 months and 12 months and remained significantly lower vs. baseline at 24 months.<br>Non-motor symptoms scale (NMSS) total score had a mean reduction at 24 months of -18.29 as compared to baseline. QoL, as assessed by PDQ-8 significantly improved at 6 months as compared to baseline and maintained its statistical significance until the 12 months evaluation. Adverse drug reactions possibly or probably related to the LCIG therapy were reported for 2.9% of the patients during the temporary naso-jejunal tube therapy and for 60.0% of the patients during permanent tube phase.

Table S4. Epidemiological studies.

Study	Region	Topic	Objective	Type of study	Method	Results
Dick 2007 a	Multicenter, international, Bucharest	Gene - Environment Interactions in Parkinsonism and PD	To investigate associations of PD and parkinsonian syndromes with polymorphic genes that influence metabolism of either foreign chemical substances or dopamine and to seek evidence of gene-environment interaction effects that modify risk.	Case-control	959 prevalent cases of parkinsonism (767 with PD) and 1989 controls across five European centers. Occupational hygienists estimated the average annual intensity of exposure to solvents, pesticides and metals, (iron, copper, manganese), blind to disease status. CYP2D6, PON1, GSTM1, GSTT1, GSTM3, GSTP1, NQOl, CYP1B1, MAO-A, MAO-B, SOD 2, EPHX, DAT1, DRD2 and NAT2 were genotyped.	There was a modest but significant association between MAO-A polymorphism in males and disease risk. The majority of gene-environment analyses did not show significant interaction effects. There were possible interaction effects between GSTM1 null genotype and solvent exposure (which were stronger when limited to PD cases only).<br>There were no specific data on Romanian participants.
Dick 2007 b	Multicenter, international, Bucharest	Environmental risk factors	To investigate the associations between PD and other degenerative parkinsonian syndromes and environmental factors	Case-control	959 prevalent cases of parkinsonism (767 with PD) and 1989 controls in Scotland, Italy, Sweden, Romania and Malta.<br>Assessment: an interviewer-administered questionnaire about lifetime occupational and hobby exposure to solvents, pesticides, iron, copper and manganese. Lifetime and average annual exposures were estimated blind to disease status using a job-exposure matrix modified by subjective exposure modelling.	Adjusted logistic regression analyses showed significantly increased odds ratios (OR) for PD/ parkinsonism with an exposure-response relationship for pesticides (low vs no exposure, OR = 1.13, 95% CI 0.82-1.57, high vs no exposure, OR = 1.41, 95% CI 1.06-1.88) and ever knocked unconscious (once vs never, OR= 1.35, 95% CI 1.09-1.68, more than once vs never, OR = 2.53, 95% CI 1.78-3.59). Hypnotic, anxiolytic or antidepressant drug use for more than 1 year and a family history of PD showed significantly increased OR. Tobacco use was protective. Analyses confined to subjects with PD gave similar results.<br>There were no specific data on Romanian participants.
De Palma 2010	Multicenter, international, Bucharest	Tobacco use	To explore the possible contribution of interactions among host and environmental factors in sporadic PD.	Retrospective, case-control	767 cases of PD and 1989 controls. Participants completed an interviewer-administered questionnaire including the history of smoking habits. The polymorphisms of genes involved either in metabolism of compounds contained in tobacco smoke (CYP2D6, CYP1B1, GSTM1, GSTT1, GSTM3, GSTP1, NQO1, SOD2, EPHX and NAT2) or in dopaminergic neurotransmission (MAOA, MAOB, DAT1 and DRD2) were characterized by PCR based methods on genomic DNA	Authors found evidence of statistically significant gene-tobacco interaction for GSTM1, NAT2, and GSTP1, the negative association between tobacco smoking and PD being significantly enhanced in subjects expressing GSTM1-1 activity, in NAT2 fast acetylators, and in those with the GSTP1*B*C haplotype.<br>There is no specific data for Romania.

Naghavi 2015	International	Age–sex specific all-cause and cause-specific mortality	To estimate yearly deaths for 188 countries between 1990, and 2013	Systematic analysis	Authors estimated age-sex-specific all-cause mortality.<br>They used six different modelling strategies across the 240 causes.<br>For all quantities reported, authors computed 95% uncertainty intervals (UIs).	Globally, the all-ages deaths in PD: 43.7 (38.3 to 55.1) thousands (in 1990), 102.5 (79.3 to 112.6) thousands (in 2013), with a Median percent change of 139.8 (77.36 to 156.99)<br>Mortality rates PD increased by 28.2% (–6.42 to 37.83).<br>The 10 leading causes of death in Romania were: ischemic heart disease, stroke, cirrhosis, lung cancer, lower respiratory infections, hypertensive heart disease, Alzheimer's disease, cardiomyopathies, COPD, colorectal cancer.<br>There was no specific data on PD in Romania.
Hay 2017	International	Disability-adjusted life-years (DALYs)	To investigate the global, regional, and national disability-adjusted life-years (DALYs) for 333 diseases and injuries and healthy life expectancy (HALE) for 195 countries and territories (1990–2016)	Systematic analysis	We calculated DALYs by summing years of life lost and years of life lived with disability for each location, age group, sex, and year. We estimated HALE using age-specific death rates and years of life lived with disability per capita. We explored how DALYs and HALE differed from expected trends when compared with the SDI: the geometric mean of income per person, educational attainment in the population older than age 15 years, and total fertility rate.	Globally, for PD, the all-age DALYs were 1304.3 (1024.7 to 1606.9) thousands (in 1090), 2385.3 (1901.1 to 2910.3) (in 2006), and 3234.5 (2563.6 to 4012.8) (in 2016)<br>The 10 leading ten causes of all-age DALYs in Romania: ischemic heart disease, stroke, low back and neck pain, falls, sense, lung cancer, migraine, hypertensive heart disease, lower respiratory infections, and road injuries.<br>Authors did not report any specific data on PD in Romania.
Naghavi 2017	International	Cause-specific deaths and years of life lost (YLLs)	To provide a comprehensive assessment of cause-specific mortality for 264 causes in 195 locations from 1980 to 2016.	Systematic analysis	Authors estimated cause-specific deaths and years of life lost (YLLs) by age, sex, geography, and year. YLLs were calculated from the sum of each death multiplied by the standard life expectancy at each age.	Globally, the number of all age deaths due to PD were: 211.3 (167.8 to 265·2) thousands (in 2016), with a percentage change from 2006 to 2016 of 40.1 (36.6 to 43.6). The all age YLLs was 2528.1 (1992.3 to 3147.4) thousands (in 2016) with a percentage change from 2006 to 2016 of 35.4 (32.4 to 38.5).<br>Two causes had statistically significant, positive annualized rates of change in age-standardized YLL rates since 2006: dengue (3.8% [95% UI 1.4–6.4]); and PD (0.25% [0.054–0.46]).<br>In Romania, the 10 leading causes of total YLLs were: ischemic heart disease, stroke, lung cancer, lower respiratory tract infections, hypertensive heart disease, cardiomyopathy and myocarditis, colorectal cancer, COPD, cirrhosis and other chronic liver diseases due to alcohol use, and Alzheimer's disease.<br>Authors did not report any specific data on PD in Romania.
Vos 2017	International	Incidence, prevalence,	To assess the prevalence, incidence, and	Systematic analysis	Authors estimated prevalence and incidence for 328 diseases and inju-	Globally, in 2016, for PD authors found a prevalence of 6063 (95% UI 4972 to 7325) thousands, an incidence of 696 (95% UI 564 to 850) thou-

		and years lived with disability (YLDs)	YLDs for 328 causes in 195 countries and territories from 1990 to 2016.		ries and 2982 sequelae, their non-fatal consequences.<br>YLDs were estimated as the product of prevalence and a disability weight for all mutually exclusive sequelae, corrected for comorbidity and aggregated to cause level.	sands, and 706 (95% 457 to 974) thousands YLDs in thousands. The percentage change in counts between 2006 and 2016 was 36.3 (95% UI 33.6 to 38.6).<br>In Romania, the 10-leading cause of YLDs were: low back pain, falls, migraine, hearing problems, neck pain, ischemic stroke, osteoarthritis, major depressive disorder, anxiety disorders, and iron deficiency anemia<br>Authors did not report any specific data on PD in Romania.
Dorsey 2018	International	Global, regional, and national burden of PD	To determine the global burden of PD between 1990 and 2016; to identify trends and to enable appropriate public health, medical, and scientific responses	Systematic analysis	Authors estimated global, regional, and country-specific prevalence and years of life lived with disability for PD from 1990 to 2016.	In 2016, 6.1 million (95% UI 5.0–7.3) individuals had PD globally, compared with 2.5 million (2.0–3.0) in 1990.<br>PD caused 3.2 million (95% UI 2.6–4.0) DALYs and 211 296 deaths (95% UI 167771–265160) in 2016. The male-to-female ratios of age standardized prevalence rates were similar in 2016 (1.40, 95% UI 1.6–1.43) and 1990 (1.37, 1.34–1.40). From 1990 to 2016, age-standardized prevalence, DALY rates, and death rates increased for all global burden of disease regions except for southern Latin America, eastern Europe, and Oceania. In addition, age-standardized DALY rates generally increased across the Socio-demographic Index.<br>Romania (2016): 1605 (1201 to 2110) deaths; 40517 (31427 to 50995) patients; 23144 (17467 to 30057) DALYs
Brodszky 2019	Central and Eastern European countries	Cost-of-illness (COI)	To provide a general description about published COI studies from CEE	Systematic search	A systematic search was performed between 1 January 2006 and 1 June 2017 in Medline, EMBASE, The Cochrane Library, CINAHL, and Web of Science to identify all relevant COI studies from nine CEE countries.	Authors identified 58 studies providing 83 country-specific COI results: Austria (n = 9), Bulgaria (n = 16), Croatia (n = 3), the Czech Republic (n = 10), Hungary (n = 24), Poland (n = 11), Romania (n = 3), Slovakia (n = 3), and Slovenia (n = 4).<br>The neurological disorders comprised 11% of the reported pathology. COI studies varied considerably in terms of methodology, publication practice, and clinical areas. Due to these heterogeneities, transferability of the COI results is limited across CEE countries.<br>Three unique studies in three different countries were conducted in PD: Austria, the Czech Republic, and Hungary. No study was conducted in Romania.
Canevelli 2019	28 countries of the European Union (EU-28) and 4 countries belonging to the European Free Trade Association (i.e., Iceland,	PD cases in migrant subjects	To estimate the number of PD cases in migrant subjects living in Europe.	Incidence	The estimated cases of PD among ≥ 50-year-old migrants living in Europe, and in each of the 32 considered countries, were calculated by multiplying the number of migrants (derived by the Eurostat data) with the age-specific prevalence rates of PD.<br>Data were updated to 2017	Nearly 20 million migrants ≥ 50 years lived in Europe in 2017. The application of the age-specific prevalence rates led to the estimation of 129,645 overall PD cases in this population, accounting for the 8% of overall PD cases in Europe.<br>National estimates widely ranged from 36 cases in Iceland to 29,390 cases in France.<br>Authors reported 665 estimated cases of PD among 69,157 migrants living in Romania

	Liechtenstein, Norway, and Switzerland).					
Feigin 2019	International	Global, regional, and national burden	To provide the most comprehensive and up-to-date estimates of the global, regional, and national burden from neurological disorders.	Systematic analysis	Authors estimated prevalence, incidence, deaths, and disability-adjusted life-years (DALYs; the sum of years of life lost [YLLs] and years lived with disability [YLDs]) by age and sex for 15 neurological disorder categories (including PD) in 195 countries from 1990 to 2016.<br>Authors quantified the contribution of 84 risks and combinations of risk to the disease estimates for the 15 neurological<br>disorder categories using the GBD comparative risk assessment approach.	PD: in 2016, 211 (168 to 265) thousands of deaths, 3235 (2564 to 4013) thousands DALYs, with a prevalence of 6063 (4971 to 7325) thousands.<br>Romania (all neurological disorders):1352 (1226 to 1487) thousands DALYs (in 1990), and 1231 (1096 to 1376) thousands DALYs (in 2016).<br>There is no specific data on PD in Romania.

Table S5. Identified research gaps and implications for research.

Identified gap	Possible Causes	Specific action to be taken	General actions to be taken
The research is focused mostly on clinical aspects of PD	This type of observational studies presents fewer technical challenges and costs	Building the necessary infrastructure for other types of research	• Increase the involvement of healthcare policy-makers and public awareness on PD<br>• Educational activities, including research methodology, and support for grant writing initiatives<br>• Increase of funding<br>• Training young specialists with a special interest in movement disorders<br>• Interdisciplinary collaboration<br>• Qualitative studies for assessing barriers and facilitators for the use of diagnostic tools and therapeutic agents
The majority of the neuropsychological studies used a level I MDS assessment	The Level II MDS assessment is more time consuming, more expensive, requires highly trained personnel, and necessitates tests adapted for the Romanian language	Training of personnel specialized in cognitive assessment<br>Translation and adaptation to the Romanian language of different international tests	
Low number of neuroimaging studies	The access to MRI was limited for many years<br>Techniques like DaT Scan are not available	Implementation of DaT Scan<br>Training of specialized personnel	
Although participated in many international, multicenter studies, none of these studies reported specific data on Romanian patients	Lack of awareness of the importance of acquiring data from Romanian population	The Romanian researchers should be encouraged to publish data on the Romanian cohorts	
No studies on interventions like DBS or apomorphine infusion	Access to DBS is limited<br>Therapies like apomorphine infusion are not available	Training of young specialists in DBS<br>Introduction of all internationally approved therapies	
Limited number of studies on non-pharmacological treatments	Lack of healthcare personnel with specialized training in PD	Contextualization to the Romanian society and the low resource context, focusing on specific demographic features	

		(i.e., multimorbidity), activity limitations, and participation restrictions	
Lack of studies on genetic forms of PD	Limited access for genetic testing	Development of the necessary resources (human and financial) for genetic testing	
Absence of precision medicine	Limited access for genetic testing<br>Limited personnel with training in personalized medicine	Development of the necessary resources (human and financial)	
Lack of epidemiological studies	Conducting population-based studies in low- and middle- income countries may be challenging due to poor infrastructure and limited resources	Development of the necessary infrastructure<br>Active case detection<br>Epidemiologic studies	
No data on the precise number of PD patients in Romania	Lack of a specialized PD registry	Initiation of a national registry of PD patients	
The research on PD is aggregated in only a few centers	Lack of specialized training	Training of young specialists and healthcare personnel with special interest in movement disorders, including PD	
Reduced number of neurologists specializing in movement disorders			
Limited number of PD nurses and occupational therapy specialists			
